# Effect of n-3 polyunsaturated fatty acids on ischemic heart disease and cardiometabolic risk factors: a two-sample Mendelian randomization study

**DOI:** 10.1186/s12872-021-02342-6

**Published:** 2021-11-08

**Authors:** Bayi Xu, Zhixia Xu, Duanmin Xu, Xuerui Tan

**Affiliations:** 1 Department of Cardiology, First Affiliated Hospital of Shantou University Medical College, Shantou, 515041 Guangdong China; 2 Department of Medical Service, Second Affiliated Hospital of Shantou University Medical College, Shantou, 515041 Guangdong China; 3 Clinical Research Center, First Affiliated Hospital of Shantou University Medical College, Shantou, 515041 Guangdong China

**Keywords:** N-3 polyunsaturated fatty acids, Mendelian randomization, Ischemic heart disease, Cardiometabolic risk factors

## Abstract

**Background:**

The cardioprotective ability of n-3 polyunsaturated fatty acids (PUFAs) is controversial. Most studies suggest a specific role for PUFAs in cardioprotection from ischemic heart disease (IHD). However, few studies have used genetic biomarkers of n-3 PUFAs to examine their potential relationships with IHD. This study aimed to use Mendelian randomization to evaluate whether genetically-predicted n-3 PUFAs affect IHD and cardiometabolic risk factors (CRFs).

**Methods:**

Genetic variants strongly (*p* < 5 × 10^–8^) and independently (*r*^2^ > 0.1) associated with n-3 PUFAs were derived from the CHARGE Consortium (including 8,866 subjects of European ancestry) and were used as instrumental variables (IVs) for evaluating the effect of n-3 PUFAs, including α-linolenic acid (ALA), docosapentaenoic acid (DPA), docosahexaenoic acid (DHA), and eicosapentaenoic acid (EPA). Data on the associations between the IVs and IHD, myocardial infarction, and CRFs (including diabetes, lipids, blood pressure, body mass index, and waist-to-hip ratio (WHR)) were obtained from the UK Biobank SOFT CAD GWAS with the CARDIoGRAMplusC4D 1000 Genomes-based GWAS (113,937 IHD cases and 339,115 controls), the Myocardial Infarction Genetics and CARDIoGRAM Exome consortia (42,335 MI cases and 78,240 controls), the DIAbetes Genetics Replication And Meta-analysis consortium (26,676 diabetes mellitus cases and 132,532 controls), the Global Lipids Genetics Consortium (n = 196,475), the International Consortium for Blood Pressure (n = 69,395), and the meta-analysis of GWAS for body fat distribution in the UK Biobank and Genetic Investigation of Anthropometric Traits (n = 694,649).

**Results:**

Genetically-predicted higher ALA was associated with lower risk of IHD, type 2 diabetes (T2D), and lower serum lipids. The effect size per 0.05-unit increase (about 1 standard deviation) in plasma ALA level) was − 1.173 (95% confidence interval − 2.214 to − 0.133) for IHD. DPA and EPA had no association with IHD but were associated with a higher risk of T2D, higher levels of lipids or WHR. DHA had no association with IHD or CRFs.

**Conclusions:**

Our study suggests a benefit of ALA for IHD and its main risk factors. DHA, DPA, and EPA had no association with IHD but were partly associated with increasing cardiometabolic risk factors.

**Supplementary Information:**

The online version contains supplementary material available at 10.1186/s12872-021-02342-6.

## Background

N-3 polyunsaturated fatty acids (PUFAs) include plant-derived α-linolenic acid (ALA) and marine-derived docosahexaenoic acid (DHA), docosapentaenoic acid (DPA), and eicosapentaenoic acid (EPA). DHA, DPA, and EPA are long-chain n-3 fatty acids. Evidence from observational, experimental studies, and randomized controlled trials (RCT) show that n-3 PUFAs from diet or supplements confer protection against cardiovascular disease (CVD) and relevant risk factors, including cardiac death, ischemic heart disease (IHD), ischemic stroke, heart failure, and blood pressure [1, 2]. However, integrated analyses of these studies have found null, little, or inconsistent results, no matter whether in primary or in secondary prevention of CVD [3–5]. Recent evidence from high-quality large RCTs also suggests that n-3 PUFA intake probably makes little or no difference for coronary heart mortality or events [6–9]. The nutrition recommendations for n-3 PUFA supplements or seafood for cardiovascular benefits have been debated in recent years, but without consensus being reached [10]. These controversies may be confounded by background dietary consumption of fish, health status, medical treatment of IHD, socioeconomic position, lifestyle, different study populations, and different definitions of CVD and study endpoints [1, 10].

Mendelian randomization (MR) studies use germline genetic variants as intermediate instrumental variables (IVs) to assess causal relationships in a non-experimental setting. As genetic variants are determined at conception, MR studies are less susceptible to confounders than observational studies and are not affected by disease status, thereby avoiding reverse causation bias. MR studies can be regarded as “natural” RCTs and have been applied to examine genetic predisposition conferred by several genes on IHD [11]. In the present study, we conducted a two-sample MR study to assess the effect of genetically predicted n-3 PUFAs on IHD, using genetically instrumented n-3 PUFAs from previous studies and a very large case–control dataset of IHD from public consortia. In addition, cardiometabolic risk factors (CRFs) of IHD, including type 2 diabetes (T2D), hyperlipidemia, hypertension, and abdominal obesity were similarly assessed.

## Methods

### Genetic instruments for n-3 PUFAs

Genetic instruments for n-3 PUFAs were obtained from published genome-wide association studies (GWAS) conducted by the Cohorts for Heart and Aging Research in Genomic Epidemiology (CHARGE) consortium [12]. The CHARGE Consortium is a design of prospective meta-analyses of GWAS from five population-based cohorts comprising 8,866 subjects of European ancestry and can be used for proxy plasma levels of n-3 fatty acids. The different n-3 PUFAs share a common metabolic pathway and single nucleotide polymorphisms (SNPs) known to influence one n-3 PUFA typically also have strong effects on the others [12]. All SNPs chosen as IVs were associated with the relevant n-3 PUFAs (ALA, DHA, DPA, and EPA) and reached genome-wide significance (*p* < 5 × 10^–8^) (Additional file 1: Tables S1–S4). The linkage disequilibrium (LD) between instrumental SNPs was obtained using LDlink [13], a web-based LD analysis tool designed to easily query pair-wise LD between SNPs in specific population groups.

### Genetic associations with IHD, T2D, lipids, blood pressure, body mass index (BMI), and waist-to-hip ratio (WHR)

Genetic associations with IHD were obtained from the published meta-analysis of UK Biobank SOFT CAD GWAS with the CARDIoGRAMplusC4D 1000 Genomes-based GWAS and the Myocardial Infarction Genetics and CARDIoGRAM Exome, which is the most up-to-date GWAS of IHD applied to the UK Biobank and involved 113,937 IHD cases and 339,115 controls in total. The UK Biobank SOFT CAD GWAS comprised 10,801 cases and 137,371 controls, and 94% of the participants were of self-reported European ancestry [14]. SOFT CAD phenotypes in UK Biobank encompassed individuals with fatal or nonfatal myocardial infarction (MI), percutaneous transluminal coronary angioplasty (PTCA), or coronary artery bypass grafting (CABG), chronic IHD, and angina. Controls were those who were free from case status. The CARDIoGRAMplusC4D 1000 Genomes-based GWAS consortium comprised 60,801 cases and 123,504 controls and most of the participants (77%) were of European descent [15]. IHD status was determined from clinical diagnosis, medical records and self-reports of medication usage, procedures such as revascularization, and other evidence of stenosis such as from coronary angiography. The Myocardial Infarction Genetics and CARDIoGRAM Exome consortium comprised 42,335 cases and 78,240 controls and all of the participants were of European descent [16].

Genetic associations with diabetes were obtained from the DIAbetes Genetics Replication and Meta-analysis (DIAGRAM) consortium. The DIAGRAM consortium is a grouping of researchers with shared interests in performing large-scale studies to characterize the genetic basis of T2D. The stage 1 analyses comprised a total of 26,676 T2D cases and 132,532 control participants of European descent [17]. Genetic associations with lipids, including high-density lipoprotein (HDL) cholesterol, low-density lipoprotein (LDL) cholesterol, total cholesterol (TC), and triglycerides (TG), were obtained from the Global Lipids Genetics Consortium (GLGC), which included 188,577 European-ancestry individuals and 7,898 non-European ancestry individuals [18]. Genetic associations with blood pressure, systolic and diastolic blood pressure (SBP and DBP), were obtained from the International Consortium for Blood Pressure (ICBP), which included 69,395 individuals of European ancestry [19]. Genetic associations with BMI and WHR were obtained from the meta-analysis of GWAS for body fat distribution in UK Biobank and Genetic Investigation of Anthropometric Traits (GIANT) and included 694,649 individuals of European ancestry [20].

### Statistical analysis

In this study, we conducted a two-sample MR analysis using R version 4.0.3 (R Foundation for Statistical Computing, Vienna, Austria) and the R package for Mendelian randomization (version 0.5.1). The main methods included inverse-variance weighted (IVW), Mendelian randomization-Egger (MR-Egger), MR-PRESSO (Mendelian Randomization Pleiotropy Residual Sum and Outlier), and weighted median (WM) methods [21–23]. These methods have their advantages and can complement each other, providing a more reliable causal inference for our study. The MR-PRESSO method can identify and exclude SNPs that most likely display pleiotropic effects [21]. All statistical tests were two-sided, and *p* < 0.05 was considered statistically significant.

**Fig. 1:**
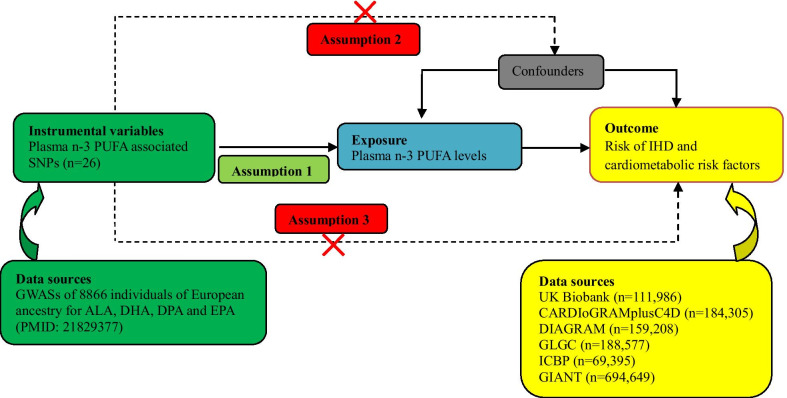
Assumptions of a Mendelian randomization analysis and data sources. Mendelian randomization analysis rests on 3 assumptions. First, the genetic instruments used as instrumental variables are strongly predictive of exposure (this study specifically refers to n-3 PUFAs, Assumption 1). Second, the association of genetic instruments with outcome (for example, IHD in this study) is not confounded by measured or unmeasured factors (Assumption 2). Third, the effect of the genetic instrument on outcome (IHD) should be fully mediated via exposure (n-3 PUFAs) and not through any direct or alternative causal pathways (Assumption 3). *ALA* α-linolenic acid, *DHA* docosahexaenoic acid, *DIAGRAM* DIAbetes Genetics Replication and Meta-analysis, *DPA* docosapentaenoic acid, *EPA* eicosapentaenoic acid, *GIANT* Genetic Investigation of Anthropometric Traits, *GLGC* Global Lipids Genetics Consortium, *GWAS* genome-wide association studies, *ICBP* International Consortium for Blood Pressure, *IHD* ischemic heart disease, *PUFAs* polyunsaturated fatty acids, *SNP* single nucleotide polymorphism

As shown in Fig. 1, MR analysis relies on three stringent assumptions [24]: (1) the genetic instruments (SNPs) used as IVs are strongly predictive of n-3 PUFA, (2) the association of genetic instruments with IHD is not confounded by measured or unmeasured factors, (3) the effect of the genetic instrument on IHD should be fully mediated via n-3 PUFAs and not through any alternative causal pathways. Pleiotropic genetic instruments violate the MR assumptions and may lead to false-positive results, interfering causal inference. We identified potential pleiotropic genetic instruments in the following ways. First, we examined the latest representative GWAS results (the DIAGRAM consortium, the GLGC consortium, the ICBP consortium, the UK Biobank, and the GIANT) to identify associations between our genetic instruments and potential biological confounding factors, including diabetes, lipids, blood pressure, BMI, and WHR. Secondly, potential pleiotropic effects of the genetic instruments were investigated by searching the literature. Genetic instruments with potential pleiotropy were excluded.

## Results

### Genetic instruments for n-3 PUFAs

After removing SNPs in linkage disequilibrium with the other SNPs (*r*^*2*^ > 0.1), 31 genome-wide significant (*p* < 5 × 10^–8^) n-3 PUFA-associated SNPs were retained for subsequent analysis (Additional file 1: Table S5). According to these 31 instrumental SNPs, genetic associations with IHD and CRFs were found from the corresponding GWAS database (Additional file 1: Tables S6–S16). Then, we examined the GWAS database to identify potential pleiotropic SNPs and found SNPs that were associated with potential confounders. Three SNPs were associated with HDL (rs174547, rs7942717, and rs174538, Additional file 1: Table S10), three SNPs were associated with TC (rs174547, rs780094, and rs174538, Additional file 1: Table S11), four SNPs were associated with TG (rs174547, rs7942717, rs780094, and rs174538, Additional file 1: Table S12), and one SNP was associated with BMI (rs780094, Additional file 1: Table S16). As pleiotropic SNPs are likely to violate the assumptions underlying MR, these SNPs were excluded from the set of IVs. No additional pleiotropic SNPs were further identified by other methods. Finally, 26 n-3 PUFA-associated SNPs were retained for IVs. These SNPs explained 0.940% (0.148–3.502%) of the variance in plasma n-3 PUFA levels (Table 1).

**Table 1:** Characteristics of the SNPs associated with plasma levels of n-3 polyunsaturated fatty acids (PUFAs)

n-3 PUFA	Nearest gene	SNP	Chr. position	EA/NEA	EAF	Effect	SE	*p*-value	VE (%)	F
ALA	FEN1	rs412334	11:61316837	t/c	0.16	− 0.0118	0.0016	9.72E−14	0.342	30
	C11orf9	rs198464	11:61278197	a/g	0.50	− 0.0057	0.0009	2.48 E−11	0.148	13
	C11orf10	rs740006	11:61314444	t/c	0.90	0.0163	0.0025	1.32 E−10	0.440	39
	C11orf9	rs17762402	11:61309777	a/g	0.07	− 0.0186	0.0029	1.56 E−10	0.411	37
DHA	ELOVL2	rs2236212	6:10995015	c/g	0.41	− 0.1132	0.0141	1.26 E−15	0.646	58
DPA	ELOVL2	rs3734398	6:10982973	t/c	0.57	− 0.0404	0.0029	9.61 E−44	2.667	243
	FEN1	rs412334	11:61560261	t/c	0.16	0.0554	0.0052	1.40 E−26	2.750	251
	C11orf10	rs740006	11:61557868	t/c	0.9	− 0.0764	0.0079	4.50 E−22	3.502	322
	FADS3	rs7394871	11:61652514	a/c	0.05	− 0.0637	0.0078	3.56 E−16	1.285	115
	FADS2	rs498793	11:61624705	t/c	0.43	0.0307	0.0038	5.84 E−16	1.540	139
	SYCP2L	rs12199131	6:10932569	a/g	0.26	0.0267	0.0033	7.69 E−16	0.914	82
	RAB3IL1	rs174472	11:61671956	a/g	0.58	0.0274	0.0037	5.74 E−14	1.219	109
	C11orf9	rs17762402	11:61553201	a/g	0.06	0.0805	0.0109	1.42 E−13	2.437	221
	SYCP2L	rs6928281	6:10908917	t/g	0.72	0.0226	0.0032	8.04 E−13	0.686	61
	C11orf9	rs198464	11:61521621	a/g	0.49	0.0191	0.0028	7.47 E−12	0.608	54
	FADS2	rs17156442	11:61614023	t/c	0.05	− 0.0513	0.0077	2.09 E−11	0.833	74
	BEST1	rs1109748	11:61722645	a/c	0.07	− 0.0400	0.0068	5.09 E−09	0.694	62
	ELOVL2	rs6936315	6:11035972	t/c	0.84	0.0244	0.0043	1.34 E−08	0.533	48
	FTH1	rs10792320	11:61746291	a/c	0.65	0.0160	0.0030	8.49 E−08	0.388	35
	BEST1	rs2727266	11:61704334	a/g	0.93	0.0308	0.0058	8.86 E−08	0.412	37
EPA	FADS3	rs7394871	11:61652514	a/c	0.05	− 0.0912	0.0128	1.13 E−12	0.494	44
	ELOVL2	rs3798713	6:11008622	c/g	0.42	0.0350	0.0050	1.93 E−12	0.373	33
	BEST1	rs1109748	11:61722645	a/c	0.07	− 0.0535	0.0092	5.46 E−09	0.233	21
	FEN1	rs412334	11:61560261	t/c	0.16	0.0440	0.0081	4.59 E−08	0.325	29
	FADS2	rs498793	11:61624705	t/c	0.43	0.0351	0.0064	5.11 E−08	0.377	34
	MAT2B	rs1145652	5:164764087	a/g	0.87	0.0356	0.0066	8.39 E−08	0.179	16

*ALA* α-linolenic acid, *Chr.* chromosome, *DHA* docosahexaenoic acid, *DPA* docosapentaenoic acid, *EA* effect allele, *EAF* effect allele frequency, *EPA* eicosapentaenoic acid, *F* F-statistic, *NEA* non-effect allele, *PUFAs* polyunsaturated fatty acids, *SE* standard error, *SNP* single nucleotide polymorphism, *VE* variation explained

VE (%) = (2 × Effect^2^ × EAF × (1 − EAF)/var (n-3 PUFAs)) × 100, var (n-3 PUFA) is the variance of n-3 PUFA and was deduced based on literature data (PMID: 27490808). F-statistic is a measure of the strength of the genetic instrument and is calculated as follows: F = (R^2^ × (n − 1 − k))/((1 − R^2^) × k), where R^2^ = VE (%), n = sample size, k = number of instrumental variables

The strength of the genetic instruments was evaluated by the F-statistic. According to methods of a previous study [25, 26], summary statistics from a previous n-3 PUFA GWAS [12, 27] were used to calculate the F-statistic. Our results showed the F-statistic of all instrumental SNPs was > 10 (mean 85, from 13 to 322) (Table 1), indicating strong genetic instruments for n-3 PUFAs [28].

### Associations of n-3 PUFAs with IHD

**Fig. 2:**
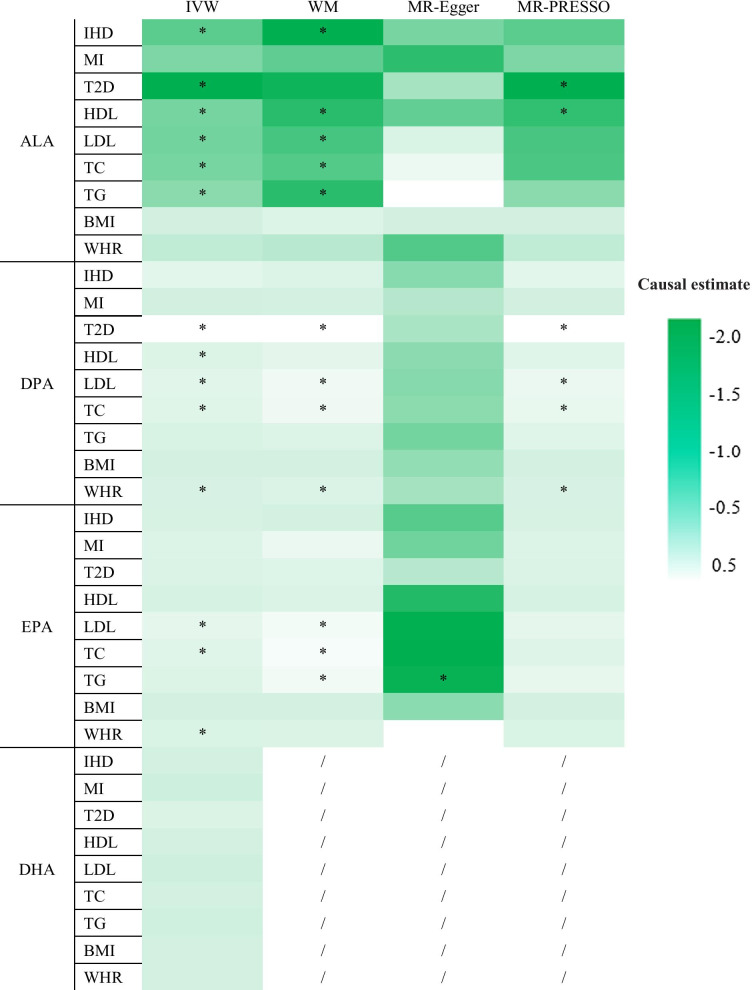
Mendelian randomization (MR) analysis testing the effects of n-3 PUFAs on IHD and cardiometabolic risk factors. Results obtained using 4 MR methods (IVW: inverse-variance weighted, WM: weighted median, MR-Egger: Mendelian randomization-Egger, and MR-PRESSO: Mendelian Randomization Pleiotropy Residual Sum and Outlier) are presented as a heat map representing causal estimates (CE). CE of SBP and DBP exceeded that of the others a lot. In order to show the others CE better, CE of SBP and DBP were not included in the heat map. *ALA* α-linolenic acid, *BMI* body mass index, *DHA* docosahexaenoic acid, *DPA* docosapentaenoic acid, *EPA* eicosapentaenoic acid, *HDL* high-density lipoprotein, *IHD* ischemic heart disease, *LDL* low-density lipoprotein, *MI* myocardial infarction, *T2D* type 2 diabetes, *TC* total cholesterol, *TG* triglycerides, *WHR* waist-to-hip ratio. *Indicates *p* < 0.05 for a particular MR approach

Figure 2 and Table S17 (Additional file 1) showed higher ALA was associated with lower IHD risk, based on IVW (*p* = 0.027) and WM (*p* = 0.006), but with inconsistent results from MR-Egger (*p* > 0.05) and MR-PRESSO (*p* > 0.05). ALA was not associated with MI, with consistent results, based on all 4 methods (IVW, WM, MR-Egger, and MR-PRESSO) (all *p* > 0.05). DPA and EPA were not significantly associated with IHD or MI, with consistent results from all 4 methods (all *p* > 0.05). MR-Egger intercepts *p* > 0.05 suggested little evidence of directional pleiotropy in all analyses. As WM, MR-Egger and MR-PRESSO methods require a minimum of 3 IVs, the associations of DHA with IHD, MI and CRFs were estimated by IVW only, and the results showed that DHA was not associated with IHD or MI (all *p* > 0.05).

### Associations of n-3 PUFAs with T2D, lipids, blood pressure, BMI, and WHR

We also tested the association of n-3 PUFAs with CRFs. As shown in Fig. 2 and Table S17 (Additional file 1), ALA was associated with a lower risk of T2D (IVW, MR-PRESSO) and lower HDL (IVW, WM, MR-PRESSO), LDL (IVW, WM), TG (IVW, WM), and TC (IVW, WM) levels (*p* < 0.05 or 0.001). ALA had no association with SBP, DBP, BMI, or WHR (all *p* > 0.05). There was little evidence of directional pleiotropy based on the MR-Egger intercept (all *p* > 0.05).

DPA was associated with a higher risk of T2D (IVW, WM, MR-PRESSO) and higher HDL (IVW), LDL (IVW, WM, MR-PRESSO), TC (IVW, WM, MR-PRESSO), and WHR (IVW, WM, MR-PRESSO) (*p* < 0.05 or 0.001). DPA had no association with TG, SBP, DBP, or BMI (all *p* > 0.05), and there was little evidence of directional pleiotropy based on the MR-Egger intercept (all *p* > 0.05). EPA was associated with higher LDL (IVW, WM), TC (IVW, WM), WHR (IVW), and lower SBP (MR-Egger), DBP (MR-Egger). As for TG, there were inconsistent results: WM indicated TG was a risk factor whereas MR-Egger indicated TG was a protective factor (both *p* < 0.05). EPA had no association with T2D, HDL, or BMI (all *p* > 0.05). Based on the MR-Egger intercept, there was potential pleiotropy between EPA and LDL (*p* = 0.021), TC (*p* = 0.044), TG (*p* = 0.003), SBP (*p* = 0.012), and DBP (*p* < 0.001). In this case, the MR-PRESSO results should prevail and we conclude that EPA had no association with LDL, TC, TG, SBP, or DBP (all *p* > 0.05). DHA had no association with CRFs.

## Discussion

This MR study showed that a genetic predisposition toward higher plasma ALA level is associated with a lower risk of IHD, but not MI. The effect size (beta coefficient) per 0.05-unit increase (about 1 SD) in plasma ALA level was − 1.173 (95% confidence interval − 2.214 to − 0.133) for IHD. In contrast, genetically-predicted levels of marine-derived n-3 PUFAs (DHA, DPA, and EPA) had no association with IHD or MI.

On primary prevention of CVD, Abdelhamid et al*.* [3] found increased ALA may slightly reduce the risk of cardiovascular events, coronary heart disease (CHD) mortality, and arrhythmia, and Pan et al*.* [29] found dietary ALA is associated with a moderately lower risk of fatal CHD, with each 1 g/d increment of ALA intake being associated with a 10% lower risk of CHD death. By assessing the primary incidence of CHD in generally healthy, free-living populations around the world, Del Gobbo et al*.* [30] also found ALA to be associated with a 9% lower risk of fatal CHD. From the data extracted from the UK Biobank SOFT CAD GWAS and the CARDIoGRAMplusC4D 1000 Genomes-based GWAS consortia, our results also demonstrate beneficial primary health outcomes for ALA. However, for secondary prevention of CVD, there is little or no effect of ALA, as previously suggested [31], or the evidence is scarce.

Our results, combined with previous findings, support the favorable effects of ALA specifically for the primary prevention of IHD. Mechanistically, these findings are supported by the effects of ALA on improving lipid profile (TC, TG, LDL) [32] and cholesterol homeostasis [33], ameliorating sympathetic heart activity and denervation [34, 35], decreasing fasting free fatty acid and inhibiting inflammation and platelet activation [36]. A meta-analysis of 18 observational studies in generally healthy populations found that ALA may be associated with modestly lower risk T2D [37]. Our results also show that genetically-predicted higher plasma ALA is associated with a lower risk of T2D and lower LDL, HDL, TG, and TC. It is well known that LDL is the initiator of IHD [38], hypertriglyceridemia is the residual risk of IHD [39, 40], while diabetes could negatively affect clinical outcomes of IHD, in patients admitted for ST-elevation myocardial infarction (STEMI) [41–43], non-STEMI [44] or stable IHD [45, 46]. From this perspective, ALA can reduce the risk of IHD in many ways. N-3 PUFAs do not affect atherosclerotic progression, plaque stability, plaque rupture, or thrombosis [10]. This may be related to the ineffectiveness of ALA on MI. Clinically, the phenotypes of MI are not equal to the presence of coronary atherosclerosis. Coronary atherosclerosis may progress as acute coronary thrombotic occlusion or MI, often due to the rupture of an unstable plaque [47–49], usually occurring in plaques with a thin, eroded fibrous cap, regardless of the degree of stenosis [47, 50]. Many patients live to advanced age with stable, significant IHD and never suffer an MI.

The clinical research on marine-derived n-3 PUFAs (DHA, DPA, and EPA) has been full of twists and turns. Before the use of statins, most of the studies on the cardioprotection of marine-derived n-3 PUFAs were positive [51]. However, after statins became widely used, most studies reported neutral effects [52, 53]. In recent years, the cardioprotective role of marine-derived n-3 PUFAs, especially EPA and DHA, has become increasingly disputed [54, 55]. Dietary recommendations of EPA and DHA have also been downgraded from Class I to Class II [53], is the reason being that a large number of more recent RCTs [6, 7] and integration analyses have found little or no effect of EPA or DHA on cardioprotection [4, 5], particularly for primary prevention of CVD [3, 8, 9]. Even when there is an effect, the effect is only seen in studies with a moderate to high risk of bias [56]. Some researchers even think fish oil has disappointing therapeutic benefits [5].

In contrast, other researchers still have hope for marine-derived n-3 PUFAs, especially EPA. They acknowledge that, in over-the-counter formulations (EPA + DHA or fish oil) at common dosages, primary prevention of CVD by marine-derived n-3 PUFAs, is ineffective and that secondary prevention is controversial [10]. They attribute the failure of the previous trials to the low dose and impure formulation of marine-derived n-3 PUFAs, short intervention duration, high background of fish intake, and inappropriate participants [51, 52, 55]. With the large success of the REDUCE-IT trial [57], proponents put their hopes on highly purified EPA (icosapent ethyl), which will lower plasma TG levels, and have given some constructive suggestions for future clinical trials [55]. At this time, the AHA also has given more affirmative recommendations to support the use of marine-derived n-3 PUFAs for reducing the residual risk of CVD that remains after statin therapy [58]. However, as fibrates [59] and PCSK9 inhibitors [60] not only reduce TG, similar to EPA, but also increase HDL and reduce LDL, a new debate arises as to whether we should use fibrates instead of EPA or PCSK9 inhibitors instead of a statin/EPA combination [61]. So, it seems that the debate on the cardioprotection of marine-derived n-3 PUFAs will continue.

Our research explores the role of marine-derived n-3 PUFAs from another perspective and finds individual marine-derived n-3 PUFAs have no association with IHD or MI in generally healthy populations. As for CRFs, DPA is associated with a higher risk of T2D and higher HDL, LDL, TC, and WHR; EPA is associated with higher WHR, and DHA does not affect CRFs. Most prior studies showed that, except for reducing TG [57, 61], marine-derived n-3 PUFAs do not affect most CRFs or intermediate outcomes [3, 9], including high CAD risk factors, *i.e.* LDL and T2D [9, 37]. Only a few studies have shown that marine-derived n-3 PUFAs significantly reduce blood pressure [62], with the greatest reductions in untreated hypertension. Some studies even suggest that marine-derived n-3 PUFAs may increase LDL [52, 58], which may negate any cardiovascular benefits [61]. Recently, a large general-practice RCT show that for patients with multiple cardiovascular risk factors (the criterion was defined as at least four of the following, or for patients with diabetes, at least one of the following: age of 65 years or older, male sex, hypertension, hypercholesterolemia, current smoker, obesity, family history of premature cardiovascular disease), treatment with n-3 PUFAs (1 g DHA + EPA daily, with a median of 5 years of follow-up) did not reduce cardiovascular mortality and morbidity [63].

There are some limitations to our study. First, in our study, both IVs and outcomes come from Europe. This avoids population stratification and conforms to the homogenous principle of an MR study [11]. However, as few n-3 PUFA GWAS are available for African Americans, Chinese, or other races, the potential effects of n-3 PUFAs by race remains to be explored. Similarly, as there are ethnic differences in the risk of cardiovascular disease [64], we need to be cautious in applying our findings to other populations. Second, n-3 PUFAs can be detected in many components of the body (e.g., serum, plasma, phospholipids, cholesterol esters, and adipose tissue) and affect many CVD subtypes (e.g., sudden cardiac death, congestive heart failure, arrhythmia, acute coronary syndrome, and stroke), but our study did not analyze this one by one. A comprehensive analysis of n-3 PUFAs in different components and their association with different disease subtypes may help to reduce potential bias and provide a better understanding of the effect of n-3 PUFAs on cardiovascular health. Third, most instrumental SNPs explain a small proportion variance of n-3 PUFAs. This may reduce the power to detect small effects of n-3 PUFAs on IHD risk in our MR framework. Fourth, the effect of n-3 PUFAs on IHD and CRFs found in this study represent a lifelong cumulative effect and are not directly comparable to those derived from conventional observational or clinical studies. Finally, we could not assess whether the effect of n-3 PUFAs on IHD and CRFs varied by sex, age, or the baseline level of n-3 PUFAs as these data are not freely available.

## Conclusions

In summary, this study indicates there are favorable effects of plant-derived ALA on IHD and CRFs, but there is no causal association between marine-derived n-3 PUFAs (DHA, DPA, EPA) and the risk of IHD. Combined with the more affordable, globally accessible, and sustainable plant sources of ALA, compared to marine-derived n-3 PUFAs, our study emphasizes the need to further explore the benefits of ALA on IHD. The benefits of marine-derived n-3 PUFAs supplements for cardioprotection remain uncertain and require testing in randomized clinical trials.

## Supplementary Information

**Additional file 1**: **Table S1.** Comprehensive results for ALA with *p* < 5 × 10^–8^. **Table S2.** Comprehensive results for DHA with *p* < 5 × 10^–8^. **Table S3.** Comprehensive results for DPA with *p* < 5 × 10^–8^. **Table S4.** Comprehensive results for EPA with *p* < 5 × 10^–8^. **Table S5.** Comprehensive results for n-3 PUFA with *p* < 5 × 10^–8^ **(**removing SNPs in linkage disequilibrium with the other SNPs). **Table S6.** Association of n-3 PUFA instrumental SNPs with IHD. **Table S7.** Association of n-3 PUFA instrumental SNPs with MI. **Table S8.** Association of n-3 PUFA instrumental SNPs with T2D. **Table S9.** Association of n-3 PUFA instrumental SNPs with LDL. **Table S10.** Association of n-3 PUFA instrumental SNPs with HDL. **Table S11.** Association of n-3 PUFA instrumental SNPs with TC. **Table S12.** Association of n-3 PUFA instrumental SNPs with TG. **Table S13.** Association of n-3 PUFA instrumental SNPs with SBP. **Table S14.** Association of n-3 PUFA instrumental SNPs with DBP. **Table S15.** Association of n-3 PUFA instrumental SNPs with WHR. **Table S16.** Association of n-3 PUFA instrumental SNPs with BMI. **Table S17.** Results of MR analyses testing causal effect of n-3 PUFA on IHD and cardiometabolic risk factors.

## Data Availability

All datasets generated and/or analyzed during this study are publicly available.

## Abbreviations

ALA: α-Linolenic acid
BMI: Body mass index
CHD: Coronary heart disease
CRFs: Cardiometabolic risk factors
DBP: Diastolic blood pressure
DHA: Docosahexaenoic acid
DPA: Docosapentaenoic acid
EPA: Eicosapentaenoic acid
GWAS: Genome-wide association studies
HDL: High-density lipoprotein
IHD: Ischemic heart disease
IVs: Instrumental variables
IVW: Inverse variance weighted
LD: Linkage disequilibrium
LDL: Low-density lipoprotein
MI: Myocardial infarction
MR: Mendelian randomization
MR-Egger: Mendelian randomization-Egger
MR-PRESSO: Mendelian Randomization Pleiotropy Residual Sum and Outlier
PUFAs: Polyunsaturated fatty acids
RCT: Randomized controlled trial
SBP: Systolic blood pressure
SNP: Single nucleotide polymorphism
STEMI: ST-elevation myocardial infarction
T2D: Type 2 diabetes
TC: Total cholesterol
TG: Triglycerides
WHR: Waist-to-hip ratio
WM: Weighted Median
