# Supplementary material for: Effect of n-3 polyunsaturated fatty acids on ischemic heart disease and cardiometabolic risk factors: a two-sample Mendelian randomization study
Source: BMC Cardiovasc Disord. 2021 Nov 8;21:532. doi: 10.1186/s12872-021-02342-6 (PMC8576934; doi:10.1186/s12872-021-02342-6)
Supplement: Supplementary file 1 — Additional file 1: Table S1. Comprehensive results for ALA with p < 5 × 10^–8^. Table S2. Comprehensive results for DHA with p < 5 × 10^–8^. Table S3. Comprehensive results for DPA with p < 5 × 10^–8^. Table S4. Comprehensive results for EPA with p < 5 × 10^–8^. Table S5. Comprehensive results for n-3 PUFA with p < 5 × 10^–8^ (removing SNPs in linkage disequilibrium with the other SNPs). Table S6. Association of n-3 PUFA instrumental SNPs with IHD. Table S7. Association of n-3 PUFA instrumental SNPs with MI. Table S8. Association of n-3 PUFA instrumental SNPs with T2D. Table S9. Association of n-3 PUFA instrumental SNPs with LDL. Table S10. Association of n-3 PUFA instrumental SNPs with HDL. Table S11. Association of n-3 PUFA instrumental SNPs with TC. Table S12. Association of n-3 PUFA instrumental SNPs with TG. Table S13. Association of n-3 PUFA instrumental SNPs with SBP. Table S14. Association of n-3 PUFA instrumental SNPs with DBP. Table S15. Association of n-3 PUFA instrumental SNPs with WHR. Table S16. Association of n-3 PUFA instrumental SNPs with BMI. Table S17. Results of MR analyses testing causal effect of n-3 PUFA on IHD and cardiometabolic risk factors. [file 12872_2021_2342_MOESM1_ESM.docx]

**Table S1.** Comprehensive results for ALA with *p* < 5×10^-8^

| **Marker Name** | **EA** | **Effect*** | **SE** | ***p* value** | **Chr. Position** | **Nearest Gene**** |
| --- | --- | --- | --- | --- | --- | --- |
| rs174547 | t | -0.0159 | 0.0009 | 3.47E-64 | 11:61327359 | **FADS1** |
| rs174550 | t | -0.0159 | 0.0009 | 5.61E-64 | 11:61328054 | **FADS1** |
| rs102275 | t | -0.0158 | 0.0009 | 7.38E-64 | 11:61314379 | **C11orf10** |
| rs174536 | a | -0.0159 | 0.0009 | 1.00E-63 | 11:61308503 | **C11orf9** |
| rs174537 | t | 0.0159 | 0.0009 | 1.04E-63 | 11:61309256 | **C11orf9** |
| rs174535 | t | -0.0159 | 0.0009 | 1.05E-63 | 11:61307932 | **C11orf9** |
| rs174545 | c | -0.0158 | 0.0009 | 1.41E-63 | 11:61325882 | **FADS1** |
| rs174546 | t | 0.0158 | 0.0009 | 1.70E-63 | 11:61326406 | **FADS1** |
| rs1535 | a | -0.0157 | 0.0009 | 3.00E-63 | 11:61354548 | **FADS2** |
| rs174574 | a | 0.0156 | 0.0009 | 2.76E-62 | 11:61356918 | **FADS2** |
| rs174577 | a | 0.0155 | 0.0009 | 1.09E-60 | 11:61361390 | **FADS2** |
| rs174576 | a | 0.0156 | 0.0009 | 1.17E-60 | 11:61360086 | **FADS2** |
| rs174583 | t | 0.0155 | 0.0009 | 2.13E-60 | 11:61366326 | **FADS2** |
| rs174541 | t | -0.0155 | 0.0009 | 3.69E-60 | 11:61322484 | FADS1 |
| rs174578 | a | 0.0155 | 0.0010 | 4.82E-60 | 11:61362075 | **FADS2** |
| rs4246215 | t | 0.0154 | 0.0009 | 8.59E-60 | 11:61320875 | **FEN1** |
| rs174528 | t | -0.0155 | 0.0010 | 3.24E-59 | 11:61300075 | **C11orf9** |
| rs174548 | c | -0.0159 | 0.0010 | 8.21E-59 | 11:61327924 | **FADS1** |
| rs174549 | a | 0.0159 | 0.0010 | 1.88E-58 | 11:61327958 | **FADS1** |
| rs174555 | t | -0.0158 | 0.0010 | 4.95E-58 | 11:61336336 | **FADS1** |
| rs174556 | t | 0.0155 | 0.0010 | 4.65E-57 | 11:61337211 | **FADS1** |
| rs174601 | t | 0.0162 | 0.0010 | 7.15E-57 | 11:61379716 | **FADS2** |
| rs174538 | a | 0.0154 | 0.0010 | 1.39E-54 | 11:61316657 | **C11orf10** |
| rs174534 | a | -0.0147 | 0.0010 | 2.70E-50 | 11:61306034 | **C11orf9** |
| rs108499 | t | 0.0148 | 0.0010 | 4.16E-50 | 11:61303813 | **C11orf9** |
| rs174570 | t | 0.0157 | 0.0013 | 9.04E-34 | 11:61353788 | **FADS2** |
| rs174575 | c | -0.0121 | 0.0010 | 8.94E-31 | 11:61358579 | **FADS2** |
| rs2727270 | t | 0.0159 | 0.0014 | 3.52E-30 | 11:61359813 | **FADS2** |
| rs2727271 | a | -0.0159 | 0.0014 | 4.97E-30 | 11:61359934 | **FADS2** |
| rs2524299 | a | -0.0155 | 0.0014 | 3.34E-29 | 11:61361358 | **FADS2** |
| rs2072114 | a | -0.015 | 0.0014 | 2.41E-28 | 11:61361791 | **FADS2** |
| rs174591 | a | 0.0116 | 0.0011 | 1.21E-26 | 11:61374252 | **FADS2** |
| rs174448 | a | -0.0094 | 0.0009 | 3.86E-25 | 11:61396149 | FADS3 |
| rs174449 | a | -0.0094 | 0.0009 | 7.61E-25 | 11:61396955 | FADS3 |
| rs2845573 | a | -0.0168 | 0.0016 | 2.79E-24 | 11:61358484 | **FADS2** |
| rs174455 | a | -0.0094 | 0.0009 | 5.90E-24 | 11:61412693 | **FADS3** |
| rs174602 | t | -0.0158 | 0.0016 | 1.82E-23 | 11:61380990 | **FADS2** |
| rs509360 | a | -0.011 | 0.0011 | 3.22E-23 | 11:61305135 | **C11orf9** |
| rs2851682 | a | -0.0162 | 0.0016 | 3.34E-23 | 11:61372588 | **FADS2** |
| rs422249 | t | 0.0094 | 0.0010 | 8.27E-23 | 11:61396064 | FADS3 |
| rs2526678 | a | 0.0172 | 0.0018 | 1.88E-22 | 11:61380369 | **FADS2** |
| rs174579 | t | 0.0106 | 0.0011 | 7.56E-21 | 11:61362189 | **FADS2** |
| rs174532 | a | -0.0114 | 0.0012 | 1.31E-20 | 11:61305450 | **C11orf9** |
| rs174593 | t | -0.0115 | 0.0013 | 1.37E-19 | 11:61375407 | **FADS2** |
| rs174616 | a | 0.0079 | 0.0009 | 1.43E-19 | 11:61385698 | **FADS2** |
| rs174597 | c | 0.0116 | 0.0013 | 1.44E-19 | 11:61377616 | **FADS2** |
| rs174585 | a | 0.0106 | 0.0012 | 1.46E-19 | 11:61368270 | **FADS2** |
| rs174611 | t | -0.0088 | 0.0010 | 2.66E-19 | 11:61384457 | **FADS2** |
| rs149803 | c | 0.0121 | 0.0014 | 8.04E-19 | 11:61295596 | **C11orf9** |
| rs174605 | t | 0.0086 | 0.0010 | 5.59E-18 | 11:61383497 | **FADS2** |
| rs174450 | t | -0.0076 | 0.0009 | 8.27E-18 | 11:61398118 | **FADS3** |
| rs174626 | a | -0.0074 | 0.0009 | 5.51E-17 | 11:61393633 | FADS2 |
| rs2269928 | t | -0.0123 | 0.0015 | 1.22E-16 | 11:61294105 | **C11orf9** |
| rs174634 | c | -0.0077 | 0.0010 | 3.20E-14 | 11:61403963 | **FADS3** |
| rs968567 | t | 0.0093 | 0.0012 | 4.43E-14 | 11:61352140 | FADS2 |
| rs174464 | a | 0.0076 | 0.0010 | 7.61E-14 | 11:61414502 | **FADS3** |
| rs412334 | t | -0.0118 | 0.0016 | 9.72E-14 | 11:61316837 | **FEN1** |
| rs174456 | a | -0.0076 | 0.0010 | 1.09E-13 | 11:61412758 | **FADS3** |
| rs1000778 | a | 0.0074 | 0.0010 | 1.13E-13 | 11:61411881 | **FADS3** |
| rs174468 | a | -0.0076 | 0.0011 | 1.11E-12 | 11:61420267 | RAB3IL1 |
| rs526126 | c | -0.0105 | 0.0015 | 1.34E-12 | 11:61381461 | **FADS2** |
| rs174478 | t | 0.0074 | 0.0011 | 3.30E-12 | 11:61435152 | **RAB3IL1** |
| rs174476 | t | -0.0074 | 0.0011 | 3.48E-12 | 11:61430694 | **RAB3IL1** |
| rs666870 | a | -0.0074 | 0.0011 | 3.54E-12 | 11:61434055 | **RAB3IL1** |
| rs174589 | c | -0.0077 | 0.0011 | 2.23E-11 | 11:61372379 | **FADS2** |
| rs198464 | a | -0.0057 | 0.0009 | 2.48E-11 | 11:61278197 | C11orf9 |
| rs198462 | a | -0.0057 | 0.0009 | 3.33E-11 | 11:61280695 | **C11orf9** |
| rs198476 | a | -0.0058 | 0.0009 | 3.56E-11 | 11:61282306 | **C11orf9** |
| rs740006 | t | 0.0163 | 0.0025 | 1.32E-10 | 11:61314444 | **C11orf10** |
| rs17762402 | a | -0.0186 | 0.0029 | 1.56E-10 | 11:61309777 | **C11orf9** |
| rs650436 | t | -0.0062 | 0.0010 | 1.87E-10 | 11:61293006 | **C11orf9** |
| rs579383 | a | 0.0058 | 0.0010 | 1.02E-09 | 11:61293159 | **C11orf9** |
| rs174627 | a | 0.0072 | 0.0012 | 2.62E-09 | 11:61394042 | FADS2 |
| rs174479 | c | -0.0089 | 0.0015 | 2.85E-09 | 11:61435330 | **RAB3IL1** |
| rs1692120 | a | -0.0051 | 0.0009 | 1.41E-08 | 11:61174048 | DAGLA |
| rs174469 | t | 0.0114 | 0.0021 | 7.47E-08 | 11:61424019 | **RAB3IL1** |
| rs2453710 | a | 0.005 | 0.0009 | 8.05E-08 | 11:61163118 | DAGLA |
| rs569258 | t | 0.0049 | 0.0009 | 8.49E-08 | 11:61277244 | C11orf9 |

*Regression coefficient associated with one copy of the effect allele.

**Nearest reference is bolded if SNP is within the reference gene.

**Table S2.** Comprehensive results for DHA with *p* <5×10^-8^

| **Marker Name** | **EA** | **Effect*** | **SE** | ***p* value** | **Chr. Position** | **Nearest Gene**** |
| --- | --- | --- | --- | --- | --- | --- |
| rs2236212 | c | -0.1132 | 0.0141 | 1.26E-15 | 6:10995015 | **ELOVL2** |
| rs3798713 | c | -0.1126 | 0.0141 | 1.40E-15 | 6:11008622 | **ELOVL2** |
| rs3734398 | t | 0.1143 | 0.0144 | 1.65E-15 | 6:10982973 | **ELOVL2** |
| rs953413 | a | -0.1108 | 0.0140 | 2.98E-15 | 6:11012859 | **ELOVL2** |
| rs4532436 | c | 0.1128 | 0.0143 | 3.17E-15 | 6:10983971 | **ELOVL2** |
| rs3798707 | t | -0.1112 | 0.0141 | 3.27E-15 | 6:10991935 | **ELOVL2** |
| rs2295602 | t | 0.1108 | 0.0141 | 3.30E-15 | 6:11005842 | **ELOVL2** |
| rs3798711 | t | 0.1107 | 0.0141 | 3.54E-15 | 6:11002810 | **ELOVL2** |
| rs1570069 | a | 0.1105 | 0.0140 | 3.61E-15 | 6:11017825 | **ELOVL2** |
| rs1225737 | t | -0.1125 | 0.0143 | 3.63E-15 | 6:10982652 | **ELOVL2** |
| rs7743830 | a | 0.1105 | 0.0140 | 3.65E-15 | 6:11014220 | **ELOVL2** |
| rs1321536 | t | 0.1106 | 0.0142 | 6.04E-15 | 6:11018812 | **ELOVL2** |
| rs9295763 | c | -0.1165 | 0.0151 | 9.87E-15 | 6:11045192 | ELOVL2 |
| rs9295764 | a | 0.1166 | 0.0151 | 1.00E-14 | 6:11045196 | ELOVL2 |
| rs1323739 | c | 0.1080 | 0.0141 | 2.00E-14 | 6:11004561 | **ELOVL2** |
| rs17675322 | a | -0.1169 | 0.0154 | 3.50E-14 | 6:11059185 | ELOVL2 |
| rs8523 | a | -0.1141 | 0.0151 | 3.94E-14 | 6:10981053 | **ELOVL2** |
| rs6900220 | t | 0.1173 | 0.0155 | 4.50E-14 | 6:11065138 | ELOVL2 |
| rs2147041 | a | 0.1169 | 0.0155 | 5.02E-14 | 6:11050523 | ELOVL2 |
| rs1225717 | a | 0.1130 | 0.0151 | 7.13E-14 | 6:10978240 | ELOVL2 |
| rs4713103 | t | -0.1129 | 0.0151 | 7.83E-14 | 6:10969141 | **SYCP2L** |
| rs7774711 | a | 0.1122 | 0.0151 | 1.25E-13 | 6:10965021 | **SYCP2L** |
| rs9295733 | a | -0.1122 | 0.0152 | 1.34E-13 | 6:10965447 | **SYCP2L** |
| rs2295600 | a | 0.1114 | 0.0152 | 1.99E-13 | 6:10962250 | **SYCP2L** |
| rs4711146 | t | 0.1114 | 0.0153 | 2.84E-13 | 6:10961405 | **SYCP2L** |
| rs6920247 | c | 0.1114 | 0.0153 | 2.92E-13 | 6:10960173 | **SYCP2L** |
| rs6918936 | c | -0.1114 | 0.0153 | 2.99E-13 | 6:10960158 | **SYCP2L** |
| rs4711171 | t | -0.1158 | 0.0160 | 4.61E-13 | 6:11074347 | HERV-FRD |
| rs1321535 | t | 0.1151 | 0.0160 | 7.12E-13 | 6:11076026 | HERV-FRD |
| rs12665478 | a | -0.1152 | 0.0160 | 7.12E-13 | 6:11080825 | HERV-FRD |
| rs4713165 | t | 0.1150 | 0.0160 | 7.23E-13 | 6:11074302 | HERV-FRD |
| rs12526913 | a | 0.1155 | 0.0161 | 7.36E-13 | 6:11082924 | HERV-FRD |
| rs4713169 | c | -0.1158 | 0.0161 | 7.48E-13 | 6:11084554 | HERV-FRD |
| rs3798721 | a | 0.1127 | 0.0161 | 2.32E-12 | 6:11040183 | **ELOVL2** |
| rs3798723 | a | -0.1129 | 0.0163 | 4.39E-12 | 6:11041720 | **ELOVL2** |
| rs9393903 | a | -0.1127 | 0.0164 | 7.29E-12 | 6:11042909 | **ELOVL2** |
| rs3798719 | t | -0.1111 | 0.0163 | 8.42E-12 | 6:11036825 | **ELOVL2** |
| rs3798722 | a | 0.1113 | 0.0164 | 1.20E-11 | 6:11040423 | **ELOVL2** |
| rs9295757 | t | -0.1105 | 0.0164 | 1.65E-11 | 6:11033625 | **ELOVL2** |
| rs1007323 | t | -0.0947 | 0.0141 | 2.20E-11 | 6:10953244 | **SYCP2L** |
| rs2180725 | t | 0.1096 | 0.0164 | 2.33E-11 | 6:11025420 | **ELOVL2** |
| rs1225741 | a | 0.0946 | 0.0142 | 2.35E-11 | 6:10952243 | **SYCP2L** |
| rs7744440 | t | 0.1081 | 0.0162 | 2.58E-11 | 6:11038511 | **ELOVL2** |
| rs17606561 | a | -0.1108 | 0.0167 | 3.43E-11 | 6:10982359 | **ELOVL2** |
| rs2295601 | a | -0.1085 | 0.0164 | 3.51E-11 | 6:11005686 | **ELOVL2** |
| rs3756963 | t | 0.1090 | 0.0165 | 4.07E-11 | 6:11022154 | **ELOVL2** |
| rs2281591 | a | 0.1082 | 0.0164 | 4.29E-11 | 6:10990493 | **ELOVL2** |
| rs7773173 | c | -0.0925 | 0.0141 | 5.42E-11 | 6:10956703 | **SYCP2L** |
| rs9368564 | a | 0.1144 | 0.0175 | 5.96E-11 | 6:11060283 | ELOVL2 |
| rs1225753 | a | 0.0915 | 0.0140 | 7.10E-11 | 6:10956689 | **SYCP2L** |
| rs1225744 | t | 0.0894 | 0.0139 | 1.28E-10 | 6:10954540 | **SYCP2L** |
| rs9393915 | t | -0.1126 | 0.0178 | 2.66E-10 | 6:11072322 | ELOVL2 |
| rs2327325 | a | -0.1018 | 0.0176 | 7.24E-09 | 6:10965654 | **SYCP2L** |
| rs9368506 | t | -0.1011 | 0.0175 | 7.38E-09 | 6:10958472 | **SYCP2L** |
| rs17674802 | a | -0.0995 | 0.0173 | 8.21E-09 | 6:10969519 | **SYCP2L** |
| rs9348766 | t | -0.0993 | 0.0173 | 9.03E-09 | 6:10968582 | **SYCP2L** |
| rs9791208 | t | 0.0997 | 0.0174 | 9.81E-09 | 6:10961649 | **SYCP2L** |

*Regression coefficient associated with one copy of the effect allele.

**Nearest reference is bolded if SNP is within the reference gene.

**Table S3.** Comprehensive results for DPA with *p* <5×10^-8^

| **Marker Name** | **EA** | **Effect*** | **SE** | ***p* value** | **Chr. Position** | **Nearest Gene**** |
| --- | --- | --- | --- | --- | --- | --- |
| rs174547 | t | 0.0746 | 0.0028 | 3.79E-154 | 11:61570783 | **FADS1** |
| rs174550 | t | 0.0746 | 0.0028 | 1.69E-153 | 11:61571478 | **FADS1** |
| rs174546 | t | -0.0744 | 0.0028 | 2.51E-153 | 11:61569830 | **FADS1** |
| rs102275 | t | 0.0744 | 0.0028 | 8.39E-153 | 11:61557803 | **C11orf10** |
| rs174545 | c | 0.0743 | 0.0028 | 2.42E-152 | 11:61569306 | **FADS1** |
| rs1535 | a | 0.0744 | 0.0028 | 2.55E-152 | 11:61597972 | **FADS2** |
| rs174535 | t | 0.0746 | 0.0028 | 1.46E-151 | 11:61551356 | **C11orf9** |
| rs174536 | a | 0.0745 | 0.0028 | 1.57E-151 | 11:61551927 | **C11orf9** |
| rs174574 | a | -0.0745 | 0.0028 | 1.93E-151 | 11:61600342 | **FADS2** |
| rs174576 | a | -0.0752 | 0.0029 | 9.13E-151 | 11:61603510 | **FADS2** |
| rs174537 | t | -0.0742 | 0.0028 | 2.11E-150 | 11:61552680 | **C11orf9** |
| rs174578 | a | -0.0751 | 0.0029 | 1.94E-149 | 11:61605499 | **FADS2** |
| rs174577 | a | -0.0747 | 0.0029 | 2.67E-149 | 11:61604814 | **FADS2** |
| rs174583 | t | -0.0743 | 0.0029 | 4.17E-147 | 11:61609750 | **FADS2** |
| rs174541 | t | 0.0724 | 0.0029 | 2.30E-140 | 11:61565908 | FADS1 |
| rs174549 | a | -0.0744 | 0.0030 | 1.30E-139 | 11:61571382 | **FADS1** |
| rs4246215 | t | -0.0721 | 0.0029 | 1.38E-139 | 11:61564299 | **FEN1** |
| rs174555 | t | 0.0739 | 0.0029 | 8.76E-139 | 11:61579760 | **FADS1** |
| rs174556 | t | -0.0729 | 0.0029 | 1.04E-138 | 11:61580635 | **FADS1** |
| rs174548 | c | 0.0737 | 0.0029 | 3.47E-138 | 11:61571348 | **FADS1** |
| rs174601 | t | -0.0780 | 0.0031 | 2.39E-136 | 11:61623140 | **FADS2** |
| rs174538 | a | -0.0738 | 0.0030 | 4.43E-136 | 11:61560081 | **C11orf10** |
| rs174528 | t | 0.0718 | 0.0029 | 1.13E-135 | 11:61543499 | **C11orf9** |
| rs174534 | a | 0.0702 | 0.0030 | 8.26E-122 | 11:61549458 | **C11orf9** |
| rs108499 | t | -0.0706 | 0.0030 | 2.52E-121 | 11:61547237 | **C11orf9** |
| rs174575 | c | 0.0617 | 0.0032 | 2.17E-84 | 11:61602003 | **FADS2** |
| rs174591 | a | -0.0605 | 0.0033 | 1.18E-73 | 11:61617676 | **FADS2** |
| rs2072114 | a | 0.0655 | 0.0039 | 4.45E-64 | 11:61605215 | **FADS2** |
| rs174579 | t | -0.0573 | 0.0034 | 4.08E-63 | 11:61605613 | **FADS2** |
| rs2727270 | t | -0.0658 | 0.0040 | 1.66E-61 | 11:61603237 | **FADS2** |
| rs2727271 | a | 0.0657 | 0.0040 | 2.29E-61 | 11:61603358 | **FADS2** |
| rs174448 | a | 0.0474 | 0.0029 | 2.89E-60 | 11:61639573 | FADS3 |
| rs174449 | a | 0.0472 | 0.0029 | 7.58E-60 | 11:61640379 | FADS3 |
| rs174570 | t | -0.0616 | 0.0038 | 8.12E-60 | 11:61597212 | **FADS2** |
| rs2524299 | a | 0.0643 | 0.0039 | 9.19E-60 | 11:61604782 | **FADS2** |
| rs174585 | a | -0.0579 | 0.0036 | 3.93E-59 | 11:61611694 | **FADS2** |
| rs174593 | t | 0.0616 | 0.0039 | 9.40E-57 | 11:61618831 | **FADS2** |
| rs174597 | c | -0.0618 | 0.0039 | 1.12E-56 | 11:61621040 | **FADS2** |
| rs174602 | t | 0.0784 | 0.0049 | 1.46E-56 | 11:61624414 | **FADS2** |
| rs422249 | t | -0.0482 | 0.0030 | 1.90E-56 | 11:61639488 | FADS3 |
| rs174455 | a | 0.0462 | 0.0029 | 9.33E-56 | 11:61656117 | **FADS3** |
| rs2845573 | a | 0.0697 | 0.0046 | 4.45E-51 | 11:61601908 | **FADS2** |
| rs174532 | a | 0.0594 | 0.0040 | 5.19E-51 | 11:61548874 | **C11orf9** |
| rs968567 | t | -0.0558 | 0.0037 | 6.15E-51 | 11:61595564 | FADS2 |
| rs174616 | a | -0.0414 | 0.0028 | 6.34E-50 | 11:61629122 | **FADS2** |
| rs174611 | t | 0.0455 | 0.0031 | 2.91E-48 | 11:61627881 | **FADS2** |
| rs174589 | c | 0.0508 | 0.0035 | 6.47E-48 | 11:61615803 | **FADS2** |
| rs174450 | t | 0.0405 | 0.0028 | 6.44E-47 | 11:61641542 | **FADS3** |
| rs174605 | t | -0.0452 | 0.0031 | 7.48E-47 | 11:61626921 | **FADS2** |
| rs2526678 | a | -0.0703 | 0.0050 | 1.16E-45 | 11:61623793 | **FADS2** |
| rs174626 | a | 0.0391 | 0.0028 | 5.63E-44 | 11:61637057 | FADS2 |
| rs3734398 | t | -0.0404 | 0.0029 | 9.61E-44 | 6:10982973 | **ELOVL2** |
| rs2851682 | a | 0.0640 | 0.0046 | 1.83E-43 | 11:61616012 | **FADS2** |
| rs2236212 | c | 0.0395 | 0.0029 | 2.87E-43 | 6:10995015 | **ELOVL2** |
| rs1225737 | t | 0.0396 | 0.0029 | 7.06E-43 | 6:10982652 | **ELOVL2** |
| rs4532436 | c | -0.0396 | 0.0029 | 7.78E-43 | 6:10983971 | **ELOVL2** |
| rs3798713 | c | 0.0392 | 0.0029 | 9.71E-43 | 6:11008622 | **ELOVL2** |
| rs3798707 | t | 0.0390 | 0.0028 | 1.19E-42 | 6:10991935 | **ELOVL2** |
| rs1321536 | t | -0.0392 | 0.0029 | 1.25E-42 | 6:11018812 | **ELOVL2** |
| rs3798711 | t | -0.0387 | 0.0028 | 2.47E-42 | 6:11002810 | **ELOVL2** |
| rs1570069 | a | -0.0386 | 0.0028 | 3.48E-42 | 6:11017825 | **ELOVL2** |
| rs2295602 | t | -0.0386 | 0.0028 | 3.56E-42 | 6:11005842 | **ELOVL2** |
| rs7743830 | a | -0.0386 | 0.0028 | 4.07E-42 | 6:11014220 | **ELOVL2** |
| rs953413 | a | 0.0383 | 0.0028 | 9.07E-42 | 6:11012859 | **ELOVL2** |
| rs17675322 | a | 0.0422 | 0.0031 | 3.30E-41 | 6:11059185 | ELOVL2 |
| rs2269928 | t | 0.0605 | 0.0045 | 6.19E-41 | 11:61537529 | **C11orf9** |
| rs9295763 | c | 0.0410 | 0.0031 | 6.64E-41 | 6:11045192 | ELOVL2 |
| rs9295764 | a | -0.0411 | 0.0031 | 6.88E-41 | 6:11045196 | ELOVL2 |
| rs9368564 | a | -0.0481 | 0.0036 | 1.00E-40 | 6:11060283 | ELOVL2 |
| rs6900220 | t | -0.0422 | 0.0032 | 1.21E-40 | 6:11065138 | ELOVL2 |
| rs526126 | c | 0.0620 | 0.0047 | 3.42E-40 | 11:61624885 | **FADS2** |
| rs9393915 | t | 0.0482 | 0.0036 | 6.09E-40 | 6:11072322 | ELOVL2 |
| rs174634 | c | 0.0422 | 0.0032 | 6.13E-40 | 11:61647387 | **FADS3** |
| rs149803 | c | -0.0592 | 0.0045 | 7.47E-40 | 11:61539020 | **C11orf9** |
| rs174464 | a | -0.0422 | 0.0032 | 1.65E-39 | 11:61657926 | **FADS3** |
| rs2147041 | a | -0.0415 | 0.0032 | 2.01E-39 | 6:11050523 | ELOVL2 |
| rs174456 | a | 0.0419 | 0.0032 | 2.72E-39 | 11:61656182 | **FADS3** |
| rs17606561 | a | 0.0451 | 0.0034 | 2.84E-39 | 6:10982359 | **ELOVL2** |
| rs9393903 | a | 0.0442 | 0.0034 | 3.19E-39 | 6:11042909 | **ELOVL2** |
| rs1000778 | a | -0.0415 | 0.0032 | 3.49E-39 | 11:61655305 | **FADS3** |
| rs8523 | a | 0.0397 | 0.0030 | 8.17E-39 | 6:10981053 | **ELOVL2** |
| rs9295757 | t | 0.0436 | 0.0033 | 8.58E-39 | 6:11033625 | **ELOVL2** |
| rs3798723 | a | 0.0436 | 0.0033 | 8.63E-39 | 6:11041720 | **ELOVL2** |
| rs2180725 | t | -0.0436 | 0.0033 | 9.58E-39 | 6:11025420 | **ELOVL2** |
| rs1321535 | t | -0.0423 | 0.0032 | 1.10E-38 | 6:11076026 | HERV-FRD |
| rs2281591 | a | -0.0437 | 0.0034 | 1.58E-38 | 6:10990493 | **ELOVL2** |
| rs2295601 | a | 0.0435 | 0.0034 | 1.68E-38 | 6:11005686 | **ELOVL2** |
| rs4713165 | t | -0.0421 | 0.0032 | 2.00E-38 | 6:11074302 | HERV-FRD |
| rs3798719 | t | 0.0430 | 0.0033 | 2.29E-38 | 6:11036825 | **ELOVL2** |
| rs12665478 | a | 0.0421 | 0.0033 | 2.39E-38 | 6:11080825 | HERV-FRD |
| rs12526913 | a | -0.0422 | 0.0033 | 2.63E-38 | 6:11082924 | HERV-FRD |
| rs4713169 | c | 0.0423 | 0.0033 | 2.82E-38 | 6:11084554 | HERV-FRD |
| rs7744440 | t | -0.0429 | 0.0033 | 5.26E-38 | 6:11038511 | **ELOVL2** |
| rs3756963 | t | -0.0434 | 0.0034 | 5.61E-38 | 6:11022154 | **ELOVL2** |
| rs4711171 | t | 0.0414 | 0.0032 | 2.45E-37 | 6:11074347 | HERV-FRD |
| rs509360 | a | 0.0456 | 0.0036 | 1.04E-36 | 11:61548559 | **C11orf9** |
| rs4713103 | t | 0.0389 | 0.0031 | 2.77E-36 | 6:10969141 | **SYCP2L** |
| rs3798722 | a | -0.0424 | 0.0034 | 2.98E-36 | 6:11040423 | **ELOVL2** |
| rs9295733 | a | 0.0387 | 0.0031 | 6.65E-36 | 6:10965447 | **SYCP2L** |
| rs2295600 | a | -0.0387 | 0.0031 | 7.51E-36 | 6:10962250 | **SYCP2L** |
| rs7774711 | a | -0.0386 | 0.0031 | 9.08E-36 | 6:10965021 | **SYCP2L** |
| rs6920247 | c | -0.0389 | 0.0031 | 1.13E-35 | 6:10960173 | **SYCP2L** |
| rs6918936 | c | 0.0389 | 0.0031 | 1.21E-35 | 6:10960158 | **SYCP2L** |
| rs1225717 | a | -0.0384 | 0.0031 | 1.25E-35 | 6:10978240 | ELOVL2 |
| rs4711146 | t | -0.0388 | 0.0031 | 1.32E-35 | 6:10961405 | **SYCP2L** |
| rs3798721 | a | -0.0410 | 0.0033 | 2.47E-35 | 6:11040183 | **ELOVL2** |
| rs174468 | a | 0.0424 | 0.0034 | 3.26E-35 | 11:61663691 | RAB3IL1 |
| rs174476 | t | 0.0408 | 0.0034 | 3.35E-33 | 11:61674118 | **RAB3IL1** |
| rs666870 | a | 0.0408 | 0.0034 | 3.40E-33 | 11:61677479 | **RAB3IL1** |
| rs174478 | t | -0.0406 | 0.0034 | 4.06E-33 | 11:61678576 | **RAB3IL1** |
| rs1323739 | c | -0.0335 | 0.0029 | 1.41E-31 | 6:11004561 | **ELOVL2** |
| rs174479 | c | 0.0545 | 0.0047 | 3.04E-31 | 11:61678754 | **RAB3IL1** |
| rs2327325 | a | 0.0435 | 0.0038 | 4.50E-31 | 6:10965654 | **SYCP2L** |
| rs17674802 | a | 0.0418 | 0.0037 | 4.78E-30 | 6:10969519 | **SYCP2L** |
| rs9348766 | t | 0.0418 | 0.0037 | 5.55E-30 | 6:10968582 | **SYCP2L** |
| rs174627 | a | -0.0444 | 0.0039 | 6.07E-30 | 11:61637466 | FADS2 |
| rs9791208 | t | -0.0419 | 0.0037 | 7.94E-30 | 6:10961649 | **SYCP2L** |
| rs9368506 | t | 0.0416 | 0.0037 | 3.12E-29 | 6:10958472 | **SYCP2L** |
| rs412334 | t | 0.0554 | 0.0052 | 1.40E-26 | 11:61560261 | **FEN1** |
| rs174469 | t | -0.0717 | 0.0068 | 8.14E-26 | 11:61667443 | **RAB3IL1** |
| rs7773173 | c | 0.0287 | 0.0028 | 6.21E-24 | 6:10956703 | **SYCP2L** |
| rs1225753 | a | -0.0283 | 0.0028 | 1.99E-23 | 6:10956689 | **SYCP2L** |
| rs1225744 | t | -0.0278 | 0.0028 | 3.05E-23 | 6:10954540 | **SYCP2L** |
| rs1225741 | a | -0.0281 | 0.0029 | 6.27E-23 | 6:10952243 | **SYCP2L** |
| rs1007323 | t | 0.0281 | 0.0029 | 7.68E-23 | 6:10953244 | **SYCP2L** |
| rs740006 | t | -0.0764 | 0.0079 | 4.50E-22 | 11:61557868 | **C11orf10** |
| rs7394871 | a | -0.0637 | 0.0078 | 3.56E-16 | 11:61652514 | **FADS3** |
| rs12207488 | a | 0.0274 | 0.0034 | 4.87E-16 | 6:10952336 | **SYCP2L** |
| rs9379969 | a | 0.0274 | 0.0034 | 5.39E-16 | 6:10952636 | **SYCP2L** |
| rs498793 | t | 0.0307 | 0.0038 | 5.84E-16 | 11:61624705 | **FADS2** |
| rs12199131 | a | 0.0267 | 0.0033 | 7.69E-16 | 6:10932569 | **SYCP2L** |
| rs12214825 | t | 0.0274 | 0.0034 | 1.05E-15 | 6:10933378 | **SYCP2L** |
| rs650436 | t | 0.0254 | 0.0032 | 1.11E-15 | 11:61536430 | **C11orf9** |
| rs17156426 | a | 0.0551 | 0.0070 | 5.02E-15 | 11:61609323 | **FADS2** |
| rs9393800 | a | -0.0266 | 0.0034 | 6.10E-15 | 6:10951737 | **SYCP2L** |
| rs7942717 | a | 0.0677 | 0.0087 | 8.49E-15 | 11:61647288 | **FADS3** |
| rs579383 | a | -0.0241 | 0.0031 | 1.12E-14 | 11:61536583 | **C11orf9** |
| rs17764935 | a | -0.0583 | 0.0076 | 1.17E-14 | 11:61664757 | RAB3IL1 |
| rs9461310 | a | -0.0250 | 0.0033 | 4.13E-14 | 6:10920196 | **SYCP2L** |
| rs174472 | a | 0.0274 | 0.0037 | 5.74E-14 | 11:61671956 | **RAB3IL1** |
| rs12213249 | t | 0.0255 | 0.0034 | 7.55E-14 | 6:10926942 | **SYCP2L** |
| rs9366669 | a | 0.0249 | 0.0033 | 1.10E-13 | 6:10908869 | **SYCP2L** |
| rs12200867 | c | 0.0255 | 0.0035 | 1.30E-13 | 6:10939824 | **SYCP2L** |
| rs17762402 | a | 0.0805 | 0.0109 | 1.42E-13 | 11:61553201 | **C11orf9** |
| rs2327323 | c | 0.0252 | 0.0034 | 2.26E-13 | 6:10933813 | **SYCP2L** |
| rs1578068 | t | 0.0251 | 0.0034 | 2.32E-13 | 6:10938104 | **SYCP2L** |
| rs9393804 | t | -0.0225 | 0.0031 | 2.33E-13 | 6:10956130 | **SYCP2L** |
| rs2235093 | a | 0.0263 | 0.0036 | 5.36E-13 | 11:61665122 | **RAB3IL1** |
| rs6928281 | t | 0.0226 | 0.0032 | 8.04E-13 | 6:10908917 | **SYCP2L** |
| rs4713044 | a | 0.0225 | 0.0032 | 9.07E-13 | 6:10911282 | **SYCP2L** |
| rs13966 | t | 0.0248 | 0.0035 | 9.92E-13 | 11:61664992 | **RAB3IL1** |
| rs7759825 | t | -0.0218 | 0.0031 | 1.18E-12 | 6:10956649 | **SYCP2L** |
| rs12190237 | a | -0.0228 | 0.0032 | 1.58E-12 | 6:10922638 | **SYCP2L** |
| rs198464 | a | 0.0191 | 0.0028 | 7.47E-12 | 11:61521621 | C11orf9 |
| rs198462 | a | 0.0193 | 0.0028 | 7.64E-12 | 11:61524119 | **C11orf9** |
| rs198476 | a | 0.0193 | 0.0028 | 8.38E-12 | 11:61525730 | **C11orf9** |
| rs17156442 | t | -0.0513 | 0.0077 | 2.09E-11 | 11:61614023 | **FADS2** |
| rs7935946 | t | -0.0482 | 0.0073 | 4.72E-11 | 11:61615542 | **FADS2** |
| rs9467921 | c | -0.0211 | 0.0033 | 1.43E-10 | 6:10910619 | **SYCP2L** |
| rs17675073 | a | -0.0270 | 0.0043 | 3.54E-10 | 6:11008649 | **ELOVL2** |
| rs9366664 | t | 0.0175 | 0.0028 | 4.18E-10 | 6:10892499 | **SYCP2L** |
| rs569258 | t | -0.0187 | 0.0030 | 4.37E-10 | 11:61520668 | C11orf9 |
| rs946350 | c | 0.0175 | 0.0028 | 4.39E-10 | 6:10892953 | **SYCP2L** |
| rs12195587 | a | -0.0269 | 0.0043 | 4.51E-10 | 6:10989942 | **ELOVL2** |
| rs17764324 | t | -0.0284 | 0.0047 | 1.05E-09 | 11:61635088 | FADS2 |
| rs17831757 | t | 0.0283 | 0.0047 | 1.16E-09 | 11:61635200 | FADS2 |
| rs916924 | t | 0.0469 | 0.0077 | 1.19E-09 | 11:61619181 | **FADS2** |
| rs11230815 | c | 0.0283 | 0.0047 | 1.22E-09 | 11:61636126 | FADS2 |
| rs3846851 | a | 0.0205 | 0.0034 | 1.24E-09 | 6:11036584 | **ELOVL2** |
| rs7104849 | a | 0.0281 | 0.0046 | 1.42E-09 | 11:61638044 | FADS3 |
| rs2521572 | t | -0.0497 | 0.0083 | 2.17E-09 | 11:61711475 | BEST1 |
| rs7482316 | a | 0.0275 | 0.0046 | 2.63E-09 | 11:61640198 | FADS3 |
| rs198426 | t | 0.0179 | 0.0030 | 3.22E-09 | 11:61490486 | **DAGLA** |
| rs3734397 | a | 0.0191 | 0.0033 | 4.48E-09 | 6:10982848 | **ELOVL2** |
| rs1109748 | a | -0.0400 | 0.0068 | 5.09E-09 | 11:61722645 | **BEST1** |
| rs976081 | t | 0.0187 | 0.0032 | 6.20E-09 | 6:11003875 | **ELOVL2** |
| rs780094 | t | 0.0167 | 0.0029 | 9.04E-09 | 2:27741237 | **GCKR** |
| rs6936315 | t | 0.0244 | 0.0043 | 1.34E-08 | 6:11035972 | **ELOVL2** |
| rs1260326 | t | 0.0165 | 0.0029 | 1.44E-08 | 2:27730940 | **GCKR** |
| rs695867 | a | 0.0400 | 0.0071 | 1.52E-08 | 11:61561288 | **FEN1** |
| rs1692120 | a | 0.0170 | 0.0030 | 1.54E-08 | 11:61417472 | DAGLA |
| rs6456745 | a | 0.0166 | 0.0029 | 1.54E-08 | 6:10894268 | **SYCP2L** |
| rs780093 | t | 0.0163 | 0.0029 | 2.04E-08 | 2:27742603 | **GCKR** |
| rs1359159 | c | -0.0237 | 0.0043 | 3.30E-08 | 6:10934371 | **SYCP2L** |
| rs9368453 | a | -0.0157 | 0.0029 | 5.90E-08 | 6:10894255 | **SYCP2L** |
| rs9368452 | t | -0.0157 | 0.0029 | 6.09E-08 | 6:10893679 | **SYCP2L** |
| rs9368446 | a | -0.0156 | 0.0029 | 6.56E-08 | 6:10892220 | **SYCP2L** |
| rs10792320 | a | 0.0160 | 0.0030 | 8.49E-08 | 11:61746291 | FTH1 |
| rs2727261 | t | -0.0368 | 0.0069 | 8.85E-08 | 11:61712131 | BEST1 |
| rs2727266 | a | 0.0308 | 0.0058 | 8.86E-08 | 11:61704334 | BEST1 |

*Regression coefficient associated with one copy of the effect allele.

**Nearest reference is bolded if SNP is within the reference gene.

**Table S4.** Comprehensive results for EPA with *p* <5×10^-8^

| **Marker Name** | **EA** | **Effect*** | **SE** | ***p* value** | **Chr. Position** | **Nearest Gene**** |
| --- | --- | --- | --- | --- | --- | --- |
| rs174538 | a | -0.0834 | 0.0052 | 5.37E-58 | 11:61560081 | **C11orf10** |
| rs174535 | t | 0.0822 | 0.0051 | 6.03E-58 | 11:61551356 | **C11orf9** |
| rs174536 | a | 0.0822 | 0.0051 | 6.68E-58 | 11:61551927 | **C11orf9** |
| rs174537 | t | -0.0819 | 0.0051 | 1.01E-57 | 11:61552680 | **C11orf9** |
| rs174550 | t | 0.0821 | 0.0051 | 1.08E-57 | 11:61571478 | **FADS1** |
| rs174547 | t | 0.0820 | 0.0051 | 1.83E-57 | 11:61570783 | **FADS1** |
| rs174546 | t | -0.0818 | 0.0051 | 2.68E-57 | 11:61569830 | **FADS1** |
| rs102275 | t | 0.0815 | 0.0051 | 3.79E-57 | 11:61557803 | **C11orf10** |
| rs174545 | c | 0.0817 | 0.0051 | 4.77E-57 | 11:61569306 | **FADS1** |
| rs174541 | t | 0.0812 | 0.0052 | 3.11E-55 | 11:61565908 | FADS1 |
| rs174574 | a | -0.0802 | 0.0051 | 4.03E-55 | 11:61600342 | **FADS2** |
| rs4246215 | t | -0.0811 | 0.0052 | 5.96E-55 | 11:61564299 | **FEN1** |
| rs1535 | a | 0.0798 | 0.0051 | 6.46E-55 | 11:61597972 | **FADS2** |
| rs174556 | t | -0.0812 | 0.0052 | 6.76E-55 | 11:61580635 | **FADS1** |
| rs174576 | a | -0.0806 | 0.0052 | 1.70E-54 | 11:61603510 | **FADS2** |
| rs174577 | a | -0.0804 | 0.0052 | 2.44E-54 | 11:61604814 | **FADS2** |
| rs174578 | a | -0.0806 | 0.0052 | 2.81E-54 | 11:61605499 | **FADS2** |
| rs174583 | t | -0.0804 | 0.0052 | 6.52E-54 | 11:61609750 | **FADS2** |
| rs174555 | t | 0.0812 | 0.0053 | 2.15E-53 | 11:61579760 | **FADS1** |
| rs174601 | t | -0.0871 | 0.0057 | 4.13E-53 | 11:61623140 | **FADS2** |
| rs174549 | a | -0.0811 | 0.0053 | 5.30E-53 | 11:61571382 | **FADS1** |
| rs174528 | t | 0.0787 | 0.0051 | 5.50E-53 | 11:61543499 | **C11orf9** |
| rs174548 | c | 0.0809 | 0.0053 | 7.85E-53 | 11:61571348 | **FADS1** |
| rs174534 | a | 0.0780 | 0.0051 | 3.20E-52 | 11:61549458 | **C11orf9** |
| rs108499 | t | -0.0788 | 0.0052 | 4.45E-52 | 11:61547237 | **C11orf9** |
| rs174570 | t | -0.0813 | 0.0066 | 3.49E-35 | 11:61597212 | **FADS2** |
| rs2845573 | a | 0.0930 | 0.0078 | 2.19E-32 | 11:61601908 | **FADS2** |
| rs2851682 | a | 0.0906 | 0.0077 | 2.83E-32 | 11:61616012 | **FADS2** |
| rs2526678 | a | -0.0988 | 0.0084 | 6.84E-32 | 11:61623793 | **FADS2** |
| rs174575 | c | 0.0631 | 0.0056 | 4.39E-29 | 11:61602003 | **FADS2** |
| rs2072114 | a | 0.0746 | 0.0067 | 1.42E-28 | 11:61605215 | **FADS2** |
| rs2727270 | t | -0.0762 | 0.0070 | 5.78E-28 | 11:61603237 | **FADS2** |
| rs2727271 | a | 0.0761 | 0.0069 | 6.10E-28 | 11:61603358 | **FADS2** |
| rs2524299 | a | 0.0748 | 0.0068 | 6.93E-28 | 11:61604782 | **FADS2** |
| rs174448 | a | 0.0536 | 0.0049 | 7.35E-28 | 11:61639573 | FADS3 |
| rs174449 | a | 0.0534 | 0.0049 | 1.12E-27 | 11:61640379 | FADS3 |
| rs174579 | t | -0.0620 | 0.0057 | 2.59E-27 | 11:61605613 | **FADS2** |
| rs174585 | a | -0.0636 | 0.0059 | 3.37E-27 | 11:61611694 | **FADS2** |
| rs422249 | t | -0.0547 | 0.0051 | 1.61E-26 | 11:61639488 | FADS3 |
| rs174455 | a | 0.0515 | 0.0049 | 5.37E-26 | 11:61656117 | **FADS3** |
| rs174591 | a | -0.0626 | 0.0059 | 6.18E-26 | 11:61617676 | **FADS2** |
| rs174605 | t | -0.0558 | 0.0054 | 8.23E-25 | 11:61626921 | **FADS2** |
| rs174611 | t | 0.0550 | 0.0054 | 1.03E-24 | 11:61627881 | **FADS2** |
| rs174593 | t | 0.0665 | 0.0066 | 8.36E-24 | 11:61618831 | **FADS2** |
| rs174597 | c | -0.0666 | 0.0066 | 8.72E-24 | 11:61621040 | **FADS2** |
| rs174602 | t | 0.0824 | 0.0083 | 5.27E-23 | 11:61624414 | **FADS2** |
| rs174589 | c | 0.0585 | 0.0059 | 5.88E-23 | 11:61615803 | **FADS2** |
| rs174616 | a | -0.0479 | 0.0050 | 4.99E-22 | 11:61629122 | **FADS2** |
| rs968567 | t | -0.0588 | 0.0062 | 3.79E-21 | 11:61595564 | FADS2 |
| rs174626 | a | 0.0457 | 0.0050 | 6.06E-20 | 11:61637057 | FADS2 |
| rs174450 | t | 0.0457 | 0.0051 | 4.97E-19 | 11:61641542 | **FADS3** |
| rs174532 | a | 0.0574 | 0.0066 | 4.72E-18 | 11:61548874 | **C11orf9** |
| rs526126 | c | 0.0674 | 0.0079 | 9.62E-18 | 11:61624885 | **FADS2** |
| rs174468 | a | 0.0500 | 0.0059 | 2.14E-17 | 11:61663691 | RAB3IL1 |
| rs666870 | a | 0.0491 | 0.0059 | 1.17E-16 | 11:61677479 | **RAB3IL1** |
| rs174476 | t | 0.0491 | 0.0059 | 1.21E-16 | 11:61674118 | **RAB3IL1** |
| rs174478 | t | -0.0490 | 0.0059 | 1.23E-16 | 11:61678576 | **RAB3IL1** |
| rs174464 | a | -0.0430 | 0.0055 | 5.23E-15 | 11:61657926 | **FADS3** |
| rs174456 | a | 0.0428 | 0.0055 | 5.77E-15 | 11:61656182 | **FADS3** |
| rs174634 | c | 0.0421 | 0.0054 | 5.80E-15 | 11:61647387 | **FADS3** |
| rs2269928 | t | 0.0639 | 0.0082 | 7.13E-15 | 11:61537529 | **C11orf9** |
| rs149803 | c | -0.0626 | 0.0080 | 7.24E-15 | 11:61539020 | **C11orf9** |
| rs1000778 | a | -0.0409 | 0.0055 | 6.19E-14 | 11:61655305 | **FADS3** |
| rs509360 | a | 0.0448 | 0.0060 | 1.16E-13 | 11:61548559 | **C11orf9** |
| rs174479 | c | 0.0548 | 0.0074 | 1.27E-13 | 11:61678754 | **RAB3IL1** |
| rs174627 | a | -0.0475 | 0.0067 | 9.53E-13 | 11:61637466 | FADS2 |
| rs7394871 | a | -0.0912 | 0.0128 | 1.13E-12 | 11:61652514 | **FADS3** |
| rs3798713 | c | 0.0350 | 0.0050 | 1.93E-12 | 6:11008622 | **ELOVL2** |
| rs2236212 | c | 0.0351 | 0.0050 | 1.97E-12 | 6:10995015 | **ELOVL2** |
| rs3734398 | t | -0.0352 | 0.0051 | 3.99E-12 | 6:10982973 | **ELOVL2** |
| rs1321536 | t | -0.0339 | 0.0050 | 1.07E-11 | 6:11018812 | **ELOVL2** |
| rs1323739 | c | -0.0337 | 0.0050 | 1.25E-11 | 6:11004561 | **ELOVL2** |
| rs1225737 | t | 0.0340 | 0.0050 | 1.52E-11 | 6:10982652 | **ELOVL2** |
| rs1570069 | a | -0.0333 | 0.0049 | 1.68E-11 | 6:11017825 | **ELOVL2** |
| rs7743830 | a | -0.0333 | 0.0049 | 1.72E-11 | 6:11014220 | **ELOVL2** |
| rs3798711 | t | -0.0333 | 0.0050 | 1.81E-11 | 6:11002810 | **ELOVL2** |
| rs953413 | a | 0.0332 | 0.0049 | 1.81E-11 | 6:11012859 | **ELOVL2** |
| rs2295602 | t | -0.0332 | 0.0049 | 1.83E-11 | 6:11005842 | **ELOVL2** |
| rs3798707 | t | 0.0334 | 0.0050 | 1.87E-11 | 6:10991935 | **ELOVL2** |
| rs17675322 | a | 0.0363 | 0.0054 | 1.87E-11 | 6:11059185 | ELOVL2 |
| rs4532436 | c | -0.0338 | 0.0050 | 2.06E-11 | 6:10983971 | **ELOVL2** |
| rs6900220 | t | -0.0364 | 0.0055 | 2.79E-11 | 6:11065138 | ELOVL2 |
| rs9295763 | c | 0.0351 | 0.0053 | 3.31E-11 | 6:11045192 | ELOVL2 |
| rs9295764 | a | -0.0352 | 0.0053 | 3.31E-11 | 6:11045196 | ELOVL2 |
| rs1321535 | t | -0.0371 | 0.0056 | 4.51E-11 | 6:11076026 | HERV-FRD |
| rs4713165 | t | -0.0370 | 0.0056 | 4.84E-11 | 6:11074302 | HERV-FRD |
| rs12665478 | a | 0.0370 | 0.0056 | 5.34E-11 | 6:11080825 | HERV-FRD |
| rs12526913 | a | -0.0372 | 0.0057 | 5.44E-11 | 6:11082924 | HERV-FRD |
| rs4713169 | c | 0.0372 | 0.0057 | 5.59E-11 | 6:11084554 | HERV-FRD |
| rs2147041 | a | -0.0350 | 0.0055 | 1.45E-10 | 6:11050523 | ELOVL2 |
| rs17764935 | a | -0.0810 | 0.0126 | 1.48E-10 | 11:61664757 | RAB3IL1 |
| rs4711171 | t | 0.0360 | 0.0056 | 1.56E-10 | 6:11074347 | HERV-FRD |
| rs17764324 | t | -0.0483 | 0.0077 | 3.73E-10 | 11:61635088 | FADS2 |
| rs17831757 | t | 0.0482 | 0.0077 | 3.92E-10 | 11:61635200 | FADS2 |
| rs11230815 | c | 0.0481 | 0.0077 | 4.04E-10 | 11:61636126 | FADS2 |
| rs8523 | a | 0.0334 | 0.0053 | 4.06E-10 | 6:10981053 | **ELOVL2** |
| rs7104849 | a | 0.0479 | 0.0077 | 4.59E-10 | 11:61638044 | FADS3 |
| rs9393915 | t | 0.0389 | 0.0063 | 6.55E-10 | 6:11072322 | ELOVL2 |
| rs3756963 | t | -0.0364 | 0.0059 | 8.33E-10 | 6:11022154 | **ELOVL2** |
| rs2180725 | t | -0.0361 | 0.0059 | 1.08E-09 | 6:11025420 | **ELOVL2** |
| rs9295757 | t | 0.0361 | 0.0059 | 1.14E-09 | 6:11033625 | **ELOVL2** |
| rs2295601 | a | 0.0359 | 0.0059 | 1.18E-09 | 6:11005686 | **ELOVL2** |
| rs9368564 | a | -0.0375 | 0.0062 | 1.28E-09 | 6:11060283 | ELOVL2 |
| rs17606561 | a | 0.0365 | 0.0060 | 1.35E-09 | 6:10982359 | **ELOVL2** |
| rs2281591 | a | -0.0359 | 0.0059 | 1.41E-09 | 6:10990493 | **ELOVL2** |
| rs3798719 | t | 0.0352 | 0.0058 | 1.49E-09 | 6:11036825 | **ELOVL2** |
| rs174469 | t | -0.0573 | 0.0095 | 1.63E-09 | 11:61667443 | **RAB3IL1** |
| rs7482316 | a | 0.0452 | 0.0075 | 1.87E-09 | 11:61640198 | FADS3 |
| rs9393903 | a | 0.0353 | 0.0059 | 2.25E-09 | 6:11042909 | **ELOVL2** |
| rs3798723 | a | 0.0348 | 0.0059 | 2.74E-09 | 6:11041720 | **ELOVL2** |
| rs1109748 | a | -0.0535 | 0.0092 | 5.46E-09 | 11:61722645 | **BEST1** |
| rs7744440 | t | -0.0334 | 0.0058 | 9.62E-09 | 6:11038511 | **ELOVL2** |
| rs2521572 | t | -0.0614 | 0.0108 | 1.20E-08 | 11:61711475 | BEST1 |
| rs3798721 | a | -0.0328 | 0.0058 | 1.56E-08 | 6:11040183 | **ELOVL2** |
| rs11230874 | t | 0.0670 | 0.0119 | 1.72E-08 | 11:61795586 | FTH1 |
| rs3798722 | a | -0.0327 | 0.0059 | 2.52E-08 | 6:11040423 | **ELOVL2** |
| rs6918936 | c | 0.0296 | 0.0054 | 3.34E-08 | 6:10960158 | **SYCP2L** |
| rs6920247 | c | -0.0296 | 0.0054 | 3.35E-08 | 6:10960173 | **SYCP2L** |
| rs4711146 | t | -0.0296 | 0.0054 | 3.37E-08 | 6:10961405 | **SYCP2L** |
| rs2295600 | a | -0.0295 | 0.0053 | 3.42E-08 | 6:10962250 | **SYCP2L** |
| rs4713103 | t | 0.0293 | 0.0053 | 3.76E-08 | 6:10969141 | **SYCP2L** |
| rs9295733 | a | 0.0293 | 0.0053 | 4.00E-08 | 6:10965447 | **SYCP2L** |
| rs1225717 | a | -0.0292 | 0.0053 | 4.21E-08 | 6:10978240 | ELOVL2 |
| rs412334 | t | 0.0440 | 0.0081 | 4.59E-08 | 11:61560261 | **FEN1** |
| rs7774711 | a | -0.0291 | 0.0053 | 4.61E-08 | 6:10965021 | **SYCP2L** |
| rs498793 | t | 0.0351 | 0.0064 | 5.11E-08 | 11:61624705 | **FADS2** |
| rs1145652 | a | 0.0356 | 0.0066 | 8.39E-08 | 5:164764087 | MAT2B |

*Regression coefficient associated with one copy of the effect allele.

**Nearest reference is bolded if SNP is within the reference gene.

**Table S5.** Comprehensive results for n-3 PUFA with *p* < 5× 10^-8^ **(**removing SNPs in linkage disequilibrium with the other SNPs)

| **n3 PUFA** | **Marker Name** | **Effect allele** | **Effect*** | **SE** | ***P* value** | **Chr. Position** | **Nearest Gene**** |
| --- | --- | --- | --- | --- | --- | --- | --- |
| ALA | rs412334 | t | -0.0118 | 0.0016 | 9.72E-14 | 11:61316837 | FEN1 |
|  | rs198464 | a | -0.0057 | 0.0009 | 2.48E-11 | 11:61278197 | C11orf9 |
|  | rs740006 | t | 0.0163 | 0.0025 | 1.32E-10 | 11:61314444 | C11orf10 |
|  | **rs174547** | t | -0.0159 | 0.0009 | 3.47E-64 | 11:61327359 | FADS1 |
|  | rs17762402 | a | -0.0186 | 0.0029 | 1.56E-10 | 11:61309777 | C11orf9 |
| DHA | rs2236212 | c | -0.1132 | 0.0141 | 1.26E-15 | 6:10995015 | ELOVL2 |
| DPA | rs3734398 | t | -0.0404 | 0.0029 | 9.61E-44 | 6:10982973 | ELOVL2 |
|  | rs412334 | t | 0.0554 | 0.0052 | 1.40E-26 | 11:61560261 | FEN1 |
|  | rs740006 | t | -0.0764 | 0.0079 | 4.50E-22 | 11:61557868 | C11orf10 |
|  | rs7394871 | a | -0.0637 | 0.0078 | 3.56E-16 | 11:61652514 | FADS3 |
|  | rs498793 | t | 0.0307 | 0.0038 | 5.84E-16 | 11:61624705 | FADS2 |
|  | rs12199131 | a | 0.0267 | 0.0033 | 7.69E-16 | 6:10932569 | SYCP2L |
|  | rs174472 | a | 0.0274 | 0.0037 | 5.74E-14 | 11:61671956 | RAB3IL1 |
|  | rs17762402 | a | 0.0805 | 0.0109 | 1.42E-13 | 11:61553201 | C11orf9 |
|  | rs6928281 | t | 0.0226 | 0.0032 | 8.04E-13 | 6:10908917 | SYCP2L |
|  | rs198464 | a | 0.0191 | 0.0028 | 7.47E-12 | 11:61521621 | C11orf9 |
|  | rs17156442 | t | -0.0513 | 0.0077 | 2.09E-11 | 11:61614023 | FADS2 |
|  | rs1109748 | a | -0.0400 | 0.0068 | 5.09E-09 | 11:61722645 | BEST1 |
|  | rs6936315 | t | 0.0244 | 0.0043 | 1.34E-08 | 6:11035972 | ELOVL2 |
|  | rs10792320 | a | 0.0160 | 0.0030 | 8.49E-08 | 11:61746291 | FTH1 |
|  | rs2727266 | a | 0.0308 | 0.0058 | 8.86E-08 | 11:61704334 | BEST1 |
|  | **rs174547** | t | 0.0746 | 0.0028 | 3.79E-154 | 11:61570783 | FADS1 |
|  | **rs780094** | t | 0.0167 | 0.0029 | 9.04E-09 | 2:27741237 | GCKR |
|  | **rs7942717** | a | 0.0677 | 0.0087 | 8.49E-15 | 11:61647288 | FADS3 |
| EPA | rs7394871 | a | -0.0912 | 0.0128 | 1.13E-12 | 11:61652514 | FADS3 |
|  | rs3798713 | c | 0.0350 | 0.0050 | 1.93E-12 | 6:11008622 | ELOVL2 |
|  | rs1109748 | a | -0.0535 | 0.0092 | 5.46E-09 | 11:61722645 | BEST1 |
|  | rs412334 | t | 0.0440 | 0.0081 | 4.59E-08 | 11:61560261 | FEN1 |
|  | rs498793 | t | 0.0351 | 0.0064 | 5.11E-08 | 11:61624705 | FADS2 |
|  | rs1145652 | a | 0.0356 | 0.0066 | 8.39E-08 | 5:164764087 | MAT2B |
|  | **rs174538** | a | -0.0834 | 0.0052 | 5.37E-58 | 11:61560081 | C11orf10 |

*Regression coefficient associated with one copy of the effect allele.

**Nearest reference is bolded if SNP is within the reference gene.

**Table S6.** Association of n-3 PUFA instrumental SNPs with IHD

| **n-3 PUFA** | **Marker Name** | **Chr. Position** | **EA/NEA** | **EAF** | **Effect*** | **SE** | ***p* value** | **N** |
| --- | --- | --- | --- | --- | --- | --- | --- | --- |
| ALA | rs198464 | 11:61521621 | a/g | 0.4715 | 0.0129 | 0.0082 | 1.17E-01 | 330171 |
|  | rs740006 | 11:61557868 | c/t | 0.0946 | 0.0099 | 0.0153 | 5.19E-01 | 323965 |
|  | rs17762402 | 11:61553201 | a/g | 0.0723 | 0.0375 | 0.0174 | 3.08E-02 | 321714 |
|  | rs412334 | 11:61560261 | t/c | 0.1403 | 0.0218 | 0.0133 | 1.00E-01 | 322561 |
|  | rs174547 | 11:61570783 | t/c | 0.6757 | 0.0278 | 0.0088 | 1.59E-03 | 335163 |
| DHA | rs2236212 | 6:10995015 | c/g | 0.4330 | 0.0012 | 0.0082 | 8.85E-01 | 336782 |
| DPA | rs198464 | 11:61521621 | a/g | 0.4715 | 0.0129 | 0.0082 | 1.17E-01 | 330171 |
|  | rs740006 | 11:61557868 | c/t | 0.0946 | 0.0099 | 0.0153 | 5.19E-01 | 323965 |
|  | rs17762402 | 11:61553201 | a/g | 0.0723 | 0.0375 | 0.0174 | 3.08E-02 | 321714 |
|  | rs412334 | 11:61560261 | t/c | 0.1403 | 0.0218 | 0.0133 | 1.00E-01 | 322561 |
|  | rs174547 | 11:61570783 | t/c | 0.6757 | 0.0278 | 0.0088 | 1.59E-03 | 335163 |
|  | rs10792320 | 11:61746291 | a/c | 0.6183 | 0.0159 | 0.0086 | 6.53E-02 | 335204 |
|  | rs17156442 | 11:61614023 | t/c | 0.0518 | 0.0086 | 0.0199 | 6.67E-01 | 321719 |
|  | rs6936315 | 6:11035972 | t/c | 0.8033 | 0.0101 | 0.0108 | 3.48E-01 | 336860 |
|  | rs2727266 | 11:61704334 | g/a | 0.0917 | 0.0013 | 0.0145 | 9.27E-01 | 335304 |
|  | rs6928281 | 6:10908917 | t/g | 0.6883 | 0.0056 | 0.0093 | 5.48E-01 | 330198 |
|  | rs7942717 | 11:61647288 | a/g | 0.9112 | 0.0116 | 0.0158 | 4.61E-01 | 323812 |
|  | rs12199131 | 6:10932569 | a/g | 0.2742 | 0.0019 | 0.0090 | 8.37E-01 | 336832 |
|  | rs174472 | 11:61671956 | a/g | 0.5630 | 0.0084 | 0.0088 | 3.39E-01 | 330183 |
|  | rs7394871 | 11:61652514 | c/a | 0.9284 | 0.0156 | 0.0190 | 4.09E-01 | 323812 |
|  | rs498793 | 11:61624705 | t/c | 0.4026 | 0.0172 | 0.0094 | 6.72E-02 | 328640 |
|  | rs1109748 | 11:61722645 | c/a | 0.8766 | 0.0159 | 0.0127 | 2.12E-01 | 335311 |
|  | rs780094 | 2:27741237 | t/c | 0.3826 | 0.0054 | 0.0084 | 5.19E-01 | 336732 |
|  | rs3734398 | 6:10982973 | c/t | 0.4476 | 0.0023 | 0.0083 | 7.83E-01 | 336772 |
| EPA | rs412334 | 11:61560261 | t/c | 0.1403 | 0.0218 | 0.0133 | 1.00E-01 | 322561 |
|  | rs7394871 | 11:61652514 | c/a | 0.9284 | 0.0156 | 0.0190 | 4.09E-01 | 323812 |
|  | rs498793 | 11:61624705 | t/c | 0.4026 | 0.0172 | 0.0094 | 6.72E-02 | 328640 |
|  | rs1109748 | 11:61722645 | c/a | 0.8766 | 0.0159 | 0.0127 | 2.12E-01 | 335311 |
|  | rs1145652 | 5:164764087 | g/a | 0.1308 | 0.0009 | 0.0126 | 9.42E-01 | 325529 |
|  | rs174538 | 11:61560081 | g/a | 0.7029 | 0.0285 | 0.0090 | 1.62E-03 | 335221 |
|  | rs3798713 | 6:11008622 | c/g | 0.4416 | 0.0007 | 0.0082 | 9.35E-01 | 336776 |

*Regression coefficient associated with one copy of the effect allele.

**Table S7.** Association of n-3 PUFA instrumental SNPs with MI

| **n-3 PUFA** | **Marker Name** | **Chr. Position** | **EA/NEA** | **EAF** | **Effect*** | **SE** | ***p* value** | **N (studies)** |
| --- | --- | --- | --- | --- | --- | --- | --- | --- |
| ALA | rs198464 | 11:61521621 | a/g | 0.4707 | 0.0045 | 0.0105 | 6.72E-01 | 42 |
|  | rs740006 | 11:61557868 | c/t | 0.0904 | 0.0014 | 0.0199 | 9.44E-01 | 41 |
|  | rs17762402 | 11:61553201 | a/g | 0.0642 | 0.0266 | 0.0232 | 2.50E-01 | 40 |
|  | rs412334 | 11:61560261 | t/c | 0.1240 | 0.0175 | 0.0186 | 3.47E-01 | 39 |
|  | rs174547 | 11:61570783 | c/t | 0.3085 | -0.0256 | 0.0113 | 2.37E-02 | 43 |
| DHA | rs2236212 | 6:10995015 | c/g | 0.4316 | 0.0062 | 0.0103 | 5.48E-01 | 44 |
| DPA | rs198464 | 11:61521621 | a/g | 0.4707 | 0.0045 | 0.0105 | 6.72E-01 | 42 |
|  | rs740006 | 11:61557868 | c/t | 0.0904 | 0.0014 | 0.0199 | 9.44E-01 | 41 |
|  | rs17762402 | 11:61553201 | a/g | 0.0642 | 0.0266 | 0.0232 | 2.50E-01 | 40 |
|  | rs412334 | 11:61560261 | t/c | 0.1240 | 0.0175 | 0.0186 | 3.47E-01 | 39 |
|  | rs174547 | 11:61570783 | c/t | 0.3085 | -0.0256 | 0.0113 | 2.37E-02 | 43 |
|  | rs10792320 | 11:61746291 | c/a | 0.3878 | -0.0118 | 0.0110 | 2.82E-01 | 43 |
|  | rs17156442 | 11:61614023 | t/c | 0.0526 | 0.0002 | 0.0262 | 9.92E-01 | 40 |
|  | rs6936315 | 6:11035972 | c/t | 0.1932 | -0.0102 | 0.0137 | 4.55E-01 | 44 |
|  | rs2727266 | 11:61704334 | g/a | 0.1079 | -0.0218 | 0.0184 | 2.35E-01 | 44 |
|  | rs6928281 | 6:10908917 | t/g | 0.6649 | 0.0075 | 0.0119 | 5.30E-01 | 43 |
|  | rs7942717 | 11:61647288 | a/g | 0.8671 | -0.0171 | 0.0200 | 3.94E-01 | 40 |
|  | rs12199131 | 6:10932569 | a/g | 0.2613 | 0.0019 | 0.0115 | 8.71E-01 | 44 |
|  | rs174472 | 11:61671956 | g/a | 0.4329 | -0.0096 | 0.0112 | 3.93E-01 | 43 |
|  | rs7394871 | 11:61652514 | c/a | 0.8817 | 0.0242 | 0.0256 | 3.44E-01 | 41 |
|  | rs498793 | 11:61624705 | c/t | 0.5740 | -0.0077 | 0.0119 | 5.20E-01 | 42 |
|  | rs1109748 | 11:61722645 | a/c | 0.1348 | -0.0123 | 0.0151 | 4.17E-01 | 44 |
|  | rs780094 | 2:27741237 | c/t | 0.5813 | 0.0031 | 0.0107 | 7.70E-01 | 33 |
|  | rs3734398 | 6:10982973 | c/t | 0.4545 | 0.0057 | 0.0105 | 5.88E-01 | 44 |
| EPA | rs412334 | 11:61560261 | t/c | 0.1240 | 0.0175 | 0.0186 | 3.47E-01 | 39 |
|  | rs7394871 | 11:61652514 | c/a | 0.8817 | 0.0242 | 0.0256 | 3.44E-01 | 41 |
|  | rs498793 | 11:61624705 | c/t | 0.5740 | -0.0077 | 0.0119 | 5.20E-01 | 42 |
|  | rs1109748 | 11:61722645 | a/c | 0.1348 | -0.0123 | 0.0151 | 4.17E-01 | 44 |
|  | rs1145652 | 5:164764087 | g/a | 0.1222 | 0.0201 | 0.0160 | 2.08E-01 | 42 |
|  | rs174538 | 11:61560081 | a/g | 0.2819 | -0.0270 | 0.0115 | 1.91E-02 | 44 |
|  | rs3798713 | 6:11008622 | c/g | 0.4412 | 0.0066 | 0.0103 | 5.22E-01 | 45 |

*Regression coefficient associated with one copy of the effect allele.

**Table S8.** Association of n-3 PUFA instrumental SNPs with T2D

| **n-3 PUFA** | **Marker Name** | **Chr. Position** | **EA/NEA** | **EAF** | **Effect*** | **SE** | ***P* value** | **N** |
| --- | --- | --- | --- | --- | --- | --- | --- | --- |
| ALA | rs198464 | 11:61521621 | a/g | 0.4968 | 0.0270 | 0.0130 | 2.90E-02 | 152598 |
|  | rs740006 | 11:61557868 | t/c | 0.8826 | -0.0280 | 0.0220 | 1.90E-01 | 158185 |
|  | rs17762402 | 11:61553201 | a/g | 0.0571 | 0.0240 | 0.0250 | 3.40E-01 | 158185 |
|  | rs412334 | 11:61560261 | t/c | 0.1511 | 0.0270 | 0.0180 | 1.40E-01 | 158185 |
|  | rs174547 | 11:61570783 | t/c | 0.6762 | 0.0490 | 0.0130 | 1.70E-04 | 152598 |
| DHA | rs2236212 | 6:10995015 | c/g | 0.4298 | -0.0076 | 0.0130 | 5.40E-01 | 152598 |
| DPA | rs198464 | 11:61521621 | a/g | 0.4968 | 0.0270 | 0.0130 | 2.90E-02 | 152598 |
|  | rs740006 | 11:61557868 | t/c | 0.8826 | -0.0280 | 0.0220 | 1.90E-01 | 158185 |
|  | rs17762402 | 11:61553201 | a/g | 0.0571 | 0.0240 | 0.0250 | 3.40E-01 | 158185 |
|  | rs412334 | 11:61560261 | t/c | 0.1511 | 0.0270 | 0.0180 | 1.40E-01 | 158185 |
|  | rs174547 | 11:61570783 | t/c | 0.6762 | 0.0490 | 0.0130 | 1.70E-04 | 152598 |
|  | rs10792320 | 11:61746291 | a/c | 0.6608 | 0.0064 | 0.0130 | 6.20E-01 | 152598 |
|  | rs17156442 | 11:61614023 | t/c | 0.0489 | -0.0550 | 0.0290 | 5.60E-02 | 158185 |
|  | rs6936315 | 6:11035972 | t/c | 0.8198 | 0.0310 | 0.0170 | 7.20E-02 | 152598 |
|  | rs2727266 | 11:61704334 | a/g | 0.9177 | 0.0170 | 0.0260 | 5.00E-01 | 158185 |
|  | rs6928281 | 6:10908917 | t/g | 0.6759 | 0.0025 | 0.0130 | 8.50E-01 | 158185 |
|  | rs7942717 | 11:61647288 | a/g | 0.9134 | 0.0440 | 0.0240 | 6.80E-02 | 158185 |
|  | rs12199131 | 6:10932569 | a/g | 0.2793 | 0.0140 | 0.0140 | 3.10E-01 | 152598 |
|  | rs174472 | 11:61671956 | a/g | 0.5830 | 0.0290 | 0.0130 | 2.40E-02 | 158185 |
|  | rs7394871 | 11:61652514 | a/c | 0.0620 | 0.0017 | 0.0300 | 9.60E-01 | 158185 |
|  | rs498793 | 11:61624705 | t/c | 0.4060 | 0.0180 | 0.0140 | 1.80E-01 | 158185 |
|  | rs1109748 | 11:61722645 | a/c | 0.0730 | -0.0140 | 0.0240 | 5.70E-01 | 158185 |
|  | rs780094 | 2:27741237 | t/c | 0.4161 | -0.0340 | 0.0130 | 8.20E-03 | 152599 |
|  | rs3734398 | 6:10982973 | t/c | 0.5656 | 0.0058 | 0.0130 | 6.40E-01 | 152598 |
| EPA | rs412334 | 11:61560261 | t/c | 0.1511 | 0.0270 | 0.0180 | 1.40E-01 | 158185 |
|  | rs7394871 | 11:61652514 | a/c | 0.0620 | 0.0017 | 0.0300 | 9.60E-01 | 158185 |
|  | rs498793 | 11:61624705 | t/c | 0.4060 | 0.0180 | 0.0140 | 1.80E-01 | 158185 |
|  | rs1109748 | 11:61722645 | a/c | 0.0730 | -0.0140 | 0.0240 | 5.70E-01 | 158185 |
|  | rs1145652 | 5:164764087 | a/g | 0.8724 | -0.0240 | 0.0180 | 1.70E-01 | 158186 |
|  | rs174538 | 11:61560081 | a/g | 0.2910 | -0.0440 | 0.0130 | 6.40E-04 | 158185 |
|  | rs3798713 | 6:11008622 | c/g | 0.4363 | -0.0098 | 0.0120 | 4.20E-01 | 158185 |

*Regression coefficient associated with one copy of the effect allele.

**Table S9.** Association of n-3 PUFA instrumental SNPs with LDL

| **n-3 PUFA** | **Marker Name** | **Chr. Position** | **EA/NEA** | **EAF** | **Effect*** | **SE** | ***P* value** | **N** |
| --- | --- | --- | --- | --- | --- | --- | --- | --- |
| ALA | rs198464 | 11:61521621 | a/g | 0.4828 | 0.0121 | 0.0052 | 7.56E-02 | 89855 |
|  | rs740006 | 11:61557868 | c/t | 0.1016 | 0.0128 | 0.0072 | 6.42E-02 | 157711 |
|  | rs17762402 | 11:61553201 | a/g | 0.0581 | 0.0218 | 0.0083 | 5.78E-03 | 159630 |
|  | rs412334 | 11:61560261 | t/c | 0.1464 | 0.0270 | 0.0055 | 1.07E+00 | 157927 |
|  | rs174547 | 11:61570783 | t/c | 0.6425 | 0.0505 | 0.0038 | 7.99E-03 | 170015 |
| DHA | rs2236212 | 6:10995015 | c/g | 0.4298 | 0.0055 | 0.0052 | 2.51E-01 | 89858 |
| DPA | rs198464 | 11:61521621 | a/g | 0.4828 | 0.0121 | 0.0052 | 7.56E-02 | 89855 |
|  | rs740006 | 11:61557868 | c/t | 0.1016 | 0.0128 | 0.0072 | 6.42E-02 | 157711 |
|  | rs17762402 | 11:61553201 | a/g | 0.0581 | 0.0218 | 0.0083 | 5.78E-03 | 159630 |
|  | rs412334 | 11:61560261 | t/c | 0.1464 | 0.0270 | 0.0055 | 1.07E+00 | 157927 |
|  | rs174547 | 11:61570783 | t/c | 0.6425 | 0.0505 | 0.0038 | 7.99E-03 | 170015 |
|  | rs10792320 | 11:61746291 | a/c | 0.6570 | 0.0088 | 0.0038 | 1.24E-02 | 171926 |
|  | rs17156442 | 11:61614023 | c/t | 0.9459 | 0.0204 | 0.0084 | 1.86E-02 | 167679 |
|  | rs6936315 | 6:11035972 | t/c | 0.8364 | 0.0044 | 0.0073 | 5.13E-01 | 89857 |
|  | rs2727266 | 11:61704334 | a/g | 0.9354 | 0.0229 | 0.0102 | 1.26E-02 | 89888 |
|  | rs6928281 | 6:10908917 | g/t | 0.2784 | 0.0011 | 0.0057 | 9.30E-01 | 89888 |
|  | rs7942717 | 11:61647288 | a/g | 0.9024 | 0.0230 | 0.0073 | 2.01E-03 | 171877 |
|  | rs12199131 | 6:10932569 | g/a | 0.7454 | 0.0061 | 0.0059 | 2.49E-01 | 89861 |
|  | rs174472 | 11:61671956 | a/g | 0.5567 | 0.0115 | 0.0047 | 1.45E-02 | 115982 |
|  | rs7394871 | 11:61652514 | c/a | 0.9472 | 0.0506 | 0.0149 | 2.79E-04 | 88049 |
|  | rs498793 | 11:61624705 | t/c | 0.4195 | 0.0185 | 0.0041 | 8.50E+00 | 172294 |
|  | rs1109748 | 11:61722645 | c/a | 0.9235 | 0.0213 | 0.0078 | 6.53E-03 | 156919 |
|  | rs780094 | 2:27741237 | t/c | 0.4142 | 0.0211 | 0.0037 | 1.02E+00 | 172941 |
|  | rs3734398 | 6:10982973 | c/t | 0.4340 | 0.0055 | 0.0052 | 2.60E-01 | 89713 |
| EPA | rs412334 | 11:61560261 | t/c | 0.1464 | 0.0270 | 0.0055 | 1.07E+00 | 157927 |
|  | rs7394871 | 11:61652514 | c/a | 0.9472 | 0.0506 | 0.0149 | 2.79E-04 | 88049 |
|  | rs498793 | 11:61624705 | t/c | 0.4195 | 0.0185 | 0.0041 | 8.50E+00 | 172294 |
|  | rs1109748 | 11:61722645 | c/a | 0.9235 | 0.0213 | 0.0078 | 6.53E-03 | 156919 |
|  | rs1145652 | 5:164764087 | a/g | 0.8654 | 0.0085 | 0.0078 | 2.45E-01 | 89888 |
|  | rs174538 | 11:61560081 | g/a | 0.6741 | 0.0500 | 0.0040 | 1.07E-03 | 167639 |
|  | rs3798713 | 6:11008622 | c/g | 0.4363 | 0.0053 | 0.0053 | 2.75E-01 | 86937 |

*Regression coefficient associated with one copy of the effect allele.

**Table S10.** Association of n-3 PUFA instrumental SNPs with HDL

| **n-3 PUFA** | **Marker Name** | **Chr. Position** | **EA/NEA** | **EAF** | **Effect*** | **SE** | ***P* value** | **N** |
| --- | --- | --- | --- | --- | --- | --- | --- | --- |
| ALA | rs198464 | 11:61521621 | a/g | 0.4828 | 0.0077 | 0.0048 | 7.59E-02 | 94276 |
|  | rs740006 | 11:61557868 | c/t | 0.1016 | 0.0082 | 0.0068 | 8.41E-02 | 166810 |
|  | rs17762402 | 11:61553201 | a/g | 0.0581 | 0.0295 | 0.0077 | 6.66E-05 | 173452 |
|  | rs412334 | 11:61560261 | t/c | 0.1464 | 0.0191 | 0.0052 | 1.64E-04 | 171793 |
|  | rs174547 | 11:61570783 | t/c | 0.6425 | 0.0389 | 0.0035 | **4.04E-27** | 184044 |
| DHA | rs2236212 | 6:10995015 | c/g | 0.4298 | 0.0004 | 0.0048 | 6.94E-01 | 94280 |
| DPA | rs198464 | 11:61521621 | a/g | 0.4828 | 0.0077 | 0.0048 | 7.59E-02 | 94276 |
|  | rs740006 | 11:61557868 | c/t | 0.1016 | 0.0082 | 0.0068 | 8.41E-02 | 166810 |
|  | rs17762402 | 11:61553201 | a/g | 0.0581 | 0.0295 | 0.0077 | 6.66E-05 | 173452 |
|  | rs412334 | 11:61560261 | t/c | 0.1464 | 0.0191 | 0.0052 | 1.64E-04 | 171793 |
|  | rs174547 | 11:61570783 | t/c | 0.6425 | 0.0389 | 0.0035 | **4.04E-27** | 184044 |
|  | rs10792320 | 11:61746291 | a/c | 0.6570 | 0.0102 | 0.0036 | 2.57E-03 | 185988 |
|  | rs17156442 | 11:61614023 | c/t | 0.9459 | 0.0374 | 0.0078 | 6.86E-05 | 181719 |
|  | rs6936315 | 6:11035972 | t/c | 0.8364 | 0.0087 | 0.0067 | 2.63E-01 | 94280 |
|  | rs2727266 | 11:61704334 | a/g | 0.9354 | 0.0206 | 0.0094 | 8.98E-03 | 94311 |
|  | rs6928281 | 6:10908917 | g/t | 0.2784 | 0.0006 | 0.0052 | 8.93E-01 | 94311 |
|  | rs7942717 | 11:61647288 | a/g | 0.9024 | 0.0397 | 0.0068 | **1.28E-08** | 185907 |
|  | rs12199131 | 6:10932569 | g/a | 0.7454 | 0.0036 | 0.0055 | 5.92E-01 | 94282 |
|  | rs174472 | 11:61671956 | a/g | 0.5567 | 0.0024 | 0.0044 | 7.86E-01 | 129341 |
|  | rs7394871 | 11:61652514 | c/a | 0.9472 | 0.0379 | 0.0134 | 1.04E-03 | 94311 |
|  | rs498793 | 11:61624705 | t/c | 0.4195 | 0.0099 | 0.0038 | 8.64E-03 | 186316 |
|  | rs1109748 | 11:61722645 | c/a | 0.9235 | 0.0290 | 0.0073 | 1.57E-04 | 169561 |
|  | rs780094 | 2:27741237 | c/t | 0.5858 | 0.0110 | 0.0035 | 2.67E-03 | 187006 |
|  | rs3734398 | 6:10982973 | t/c | 0.5660 | 0.0001 | 0.0048 | 6.16E-01 | 94135 |
| EPA | rs412334 | 11:61560261 | t/c | 0.1464 | 0.0191 | 0.0052 | 1.64E-04 | 171793 |
|  | rs7394871 | 11:61652514 | c/a | 0.9472 | 0.0379 | 0.0134 | 1.04E-03 | 94311 |
|  | rs498793 | 11:61624705 | t/c | 0.4195 | 0.0099 | 0.0038 | 8.64E-03 | 186316 |
|  | rs1109748 | 11:61722645 | c/a | 0.9235 | 0.0290 | 0.0073 | 1.57E-04 | 169561 |
|  | rs1145652 | 5:164764087 | g/a | 0.1346 | 0.0015 | 0.0072 | 8.88E-01 | 94311 |
|  | rs174538 | 11:61560081 | g/a | 0.6741 | 0.0332 | 0.0037 | **7.90E-20** | 181678 |
|  | rs3798713 | 6:11008622 | c/g | 0.4363 | 0.0005 | 0.0049 | 6.65E-01 | 91319 |

*Regression coefficient associated with one copy of the effect allele.

**Table S11.** Association of n-3 PUFA instrumental SNPs with TC

| **n-3 PUFA** | **Marker Name** | **Chr. Position** | **EA/NEA** | **EAF** | **Effect*** | **SE** | ***P* value** | **N** |
| --- | --- | --- | --- | --- | --- | --- | --- | --- |
| ALA | rs198464 | 11:61521621 | a/g | 0.4828 | 0.0135 | 0.0051 | 5.50E-02 | 94560 |
|  | rs740006 | 11:61557868 | c/t | 0.1016 | 0.0108 | 0.0069 | 1.05E-01 | 171095 |
|  | rs17762402 | 11:61553201 | a/g | 0.0581 | 0.0202 | 0.0079 | 2.70E-03 | 173634 |
|  | rs412334 | 11:61560261 | t/c | 0.1464 | 0.0243 | 0.0053 | 2.43E-06 | 171977 |
|  | rs174547 | 11:61570783 | t/c | 0.6425 | 0.0472 | 0.0037 | **1.35E-35** | 184184 |
| DHA | rs2236212 | 6:10995015 | c/g | 0.4298 | 0.0012 | 0.0051 | 6.77E-01 | 94562 |
| DPA | rs198464 | 11:61521621 | a/g | 0.4828 | 0.0135 | 0.0051 | 5.50E-02 | 94560 |
|  | rs740006 | 11:61557868 | c/t | 0.1016 | 0.0108 | 0.0069 | 1.05E-01 | 171095 |
|  | rs17762402 | 11:61553201 | a/g | 0.0581 | 0.0202 | 0.0079 | 2.70E-03 | 173634 |
|  | rs412334 | 11:61560261 | t/c | 0.1464 | 0.0243 | 0.0053 | 2.43E-06 | 171977 |
|  | rs174547 | 11:61570783 | t/c | 0.6425 | 0.0472 | 0.0037 | **1.35E-35** | 184184 |
|  | rs10792320 | 11:61746291 | a/c | 0.6570 | 0.0051 | 0.0037 | 1.05E-01 | 186175 |
|  | rs17156442 | 11:61614023 | c/t | 0.9459 | 0.0235 | 0.0081 | 9.08E-03 | 181903 |
|  | rs6936315 | 6:11035972 | t/c | 0.8364 | 0.0027 | 0.0072 | 6.64E-01 | 94564 |
|  | rs2727266 | 11:61704334 | a/g | 0.9354 | 0.0243 | 0.0100 | 7.00E-03 | 94595 |
|  | rs6928281 | 6:10908917 | g/t | 0.2784 | 0.0004 | 0.0055 | 8.70E-01 | 94595 |
|  | rs7942717 | 11:61647288 | a/g | 0.9024 | 0.0288 | 0.0070 | 1.02E-04 | 186095 |
|  | rs12199131 | 6:10932569 | g/a | 0.7454 | 0.0072 | 0.0058 | 2.04E-01 | 94566 |
|  | rs174472 | 11:61671956 | a/g | 0.5567 | 0.0058 | 0.0044 | 1.82E-01 | 129266 |
|  | rs7394871 | 11:61652514 | c/a | 0.9472 | 0.0449 | 0.0144 | 5.13E-04 | 94595 |
|  | rs498793 | 11:61624705 | t/c | 0.4195 | 0.0178 | 0.0039 | 3.31E-06 | 186500 |
|  | rs1109748 | 11:61722645 | c/a | 0.9235 | 0.0299 | 0.0074 | 1.48E-04 | 168859 |
|  | rs780094 | 2:27741237 | t/c | 0.4142 | 0.0504 | 0.0036 | **5.28E-41** | 187196 |
|  | rs3734398 | 6:10982973 | c/t | 0.4340 | 0.0018 | 0.0051 | 6.09E-01 | 94413 |
| EPA | rs412334 | 11:61560261 | t/c | 0.1464 | 0.0243 | 0.0053 | 2.43E-06 | 171977 |
|  | rs7394871 | 11:61652514 | c/a | 0.9472 | 0.0449 | 0.0144 | 5.13E-04 | 94595 |
|  | rs498793 | 11:61624705 | t/c | 0.4195 | 0.0178 | 0.0039 | 3.31E-06 | 186500 |
|  | rs1109748 | 11:61722645 | c/a | 0.9235 | 0.0299 | 0.0074 | 1.48E-04 | 168859 |
|  | rs1145652 | 5:164764087 | a/g | 0.8654 | 0.0096 | 0.0076 | 1.37E-01 | 94595 |
|  | rs174538 | 11:61560081 | g/a | 0.6741 | 0.0461 | 0.0038 | **2.45E-32** | 181862 |
|  | rs3798713 | 6:11008622 | c/g | 0.4363 | 0.0009 | 0.0052 | 7.43E-01 | 91559 |

*Regression coefficient associated with one copy of the effect allele.

**Table S12.** Association of n-3 PUFA instrumental SNPs with TG

| **n-3 PUFA** | **Marker Name** | **Chr. Position** | **EA/NEA** | **EAF** | **Effect*** | **SE** | ***P* value** | **N** |
| --- | --- | --- | --- | --- | --- | --- | --- | --- |
| ALA | rs198464 | 11:61521621 | g/a | 0.5172 | 0.0149 | 0.0047 | 4.54E-03 | 90978 |
|  | rs740006 | 11:61557868 | t/c | 0.8984 | 0.0229 | 0.0065 | 6.97E-05 | 162020 |
|  | rs17762402 | 11:61553201 | g/a | 0.9420 | 0.0314 | 0.0075 | 1.31E-05 | 164146 |
|  | rs412334 | 11:61560261 | c/t | 0.8536 | 0.0177 | 0.0049 | 5.95E-04 | 162481 |
|  | rs174547 | 11:61570783 | c/t | 0.3575 | 0.0469 | 0.0035 | **1.04E-40** | 174696 |
| DHA | rs2236212 | 6:10995015 | g/c | 1.0000 | 0.0040 | 0.0047 | 4.24E-01 | 90980 |
| DPA | rs198464 | 11:61521621 | g/a | 0.5172 | 0.0149 | 0.0047 | 4.54E-03 | 90978 |
|  | rs740006 | 11:61557868 | t/c | 0.8984 | 0.0229 | 0.0065 | 6.97E-05 | 162020 |
|  | rs17762402 | 11:61553201 | g/a | 0.9420 | 0.0314 | 0.0075 | 1.31E-05 | 164146 |
|  | rs412334 | 11:61560261 | c/t | 0.8536 | 0.0177 | 0.0049 | 5.95E-04 | 162481 |
|  | rs174547 | 11:61570783 | c/t | 0.3575 | 0.0469 | 0.0035 | **1.04E-40** | 174696 |
|  | rs10792320 | 11:61746291 | c/a | 0.3430 | 0.0165 | 0.0035 | 4.52E-06 | 176695 |
|  | rs17156442 | 11:61614023 | t/c | 0.0541 | 0.0416 | 0.0076 | 8.24E-06 | 172415 |
|  | rs6936315 | 6:11035972 | c/t | 0.1636 | 0.0024 | 0.0066 | 6.61E-01 | 90982 |
|  | rs2727266 | 11:61704334 | g/a | 0.0646 | 0.0249 | 0.0091 | 1.50E-02 | 91013 |
|  | rs6928281 | 6:10908917 | g/t | 0.2784 | 0.0002 | 0.0051 | 9.29E-01 | 91013 |
|  | rs7942717 | 11:61647288 | g/a | 0.0976 | 0.0393 | 0.0067 | **1.11E-08** | 176606 |
|  | rs12199131 | 6:10932569 | g/a | 0.7454 | 0.0017 | 0.0053 | 7.58E-01 | 90984 |
|  | rs174472 | 11:61671956 | a/g | 0.5567 | 0.0006 | 0.0041 | 8.48E-01 | 119928 |
|  | rs7394871 | 11:61652514 | a/c | 0.0528 | 0.0543 | 0.0132 | 1.41E-03 | 89073 |
|  | rs498793 | 11:61624705 | c/t | 0.5805 | 0.0114 | 0.0037 | 3.10E-03 | 177023 |
|  | rs1109748 | 11:61722645 | a/c | 0.0765 | 0.0188 | 0.0070 | 1.20E-03 | 160838 |
|  | rs780094 | 2:27741237 | t/c | 0.4142 | 0.1102 | 0.0034 | **2.65E-220** | 177705 |
|  | rs3734398 | 6:10982973 | t/c | 0.5660 | 0.0032 | 0.0047 | 5.12E-01 | 90831 |
| EPA | rs412334 | 11:61560261 | c/t | 0.8536 | 0.0177 | 0.0049 | 5.95E-04 | 162481 |
|  | rs7394871 | 11:61652514 | a/c | 0.0528 | 0.0543 | 0.0132 | 1.41E-03 | 89073 |
|  | rs498793 | 11:61624705 | c/t | 0.5805 | 0.0114 | 0.0037 | 3.10E-03 | 177023 |
|  | rs1109748 | 11:61722645 | a/c | 0.0765 | 0.0188 | 0.0070 | 1.20E-03 | 160838 |
|  | rs1145652 | 5:164764087 | g/a | 0.1346 | 0.0120 | 0.0069 | 2.45E-01 | 91013 |
|  | rs174538 | 11:61560081 | a/g | 0.3259 | 0.0387 | 0.0036 | **3.46E-28** | 172374 |
|  | rs3798713 | 6:11008622 | g/c | 1.0000 | 0.0044 | 0.0047 | 3.86E-01 | 87977 |

*Regression coefficient associated with one copy of the effect allele.

**Table S13.** Association of n-3 PUFA instrumental SNPs with SBP

| **n-3 PUFA** | **Marker Name** | **Chr. Position** | **EA/NEA** | **EAF** | **Effect*** | **SE** | ***p* value** | **N** |
| --- | --- | --- | --- | --- | --- | --- | --- | --- |
| ALA | rs198464 | 11:61521621 | a/g | 0.4968 | 0.0339 | 0.0973 | 7.28E-01 | 68367 |
|  | rs740006 | 11:61557868 | t/c | 0.8826 | 0.1539 | 0.2144 | 4.73E-01 | 33333 |
|  | rs17762402 | 11:61553201 | g/a | 0.0571 | 0.4377 | 0.2787 | 1.16E-01 | 27038 |
|  | rs412334 | 11:61560261 | t/c | 0.1511 | 0.1597 | 0.1729 | 3.56E-01 | 40785 |
|  | rs174547 | 11:61570783 | c/t | 0.6762 | 0.0321 | 0.1018 | 7.52E-01 | 69799 |
| DHA | rs2236212 | 6:10995015 | c/g | 0.4298 | 0.1230 | 0.0976 | 2.07E-01 | 69561 |
| DPA | rs198464 | 11:61521621 | a/g | 0.4968 | 0.0339 | 0.0973 | 7.28E-01 | 68367 |
|  | rs740006 | 11:61557868 | t/c | 0.8826 | 0.1539 | 0.2144 | 4.73E-01 | 33333 |
|  | rs17762402 | 11:61553201 | g/a | 0.0571 | 0.4377 | 0.2787 | 1.16E-01 | 27038 |
|  | rs412334 | 11:61560261 | t/c | 0.1511 | 0.1597 | 0.1729 | 3.56E-01 | 40785 |
|  | rs174547 | 11:61570783 | c/t | 0.6762 | 0.0321 | 0.1018 | 7.52E-01 | 69799 |
|  | rs10792320 | 11:61746291 | a/c | 0.6608 | 0.0813 | 0.1016 | 4.24E-01 | 68196 |
|  | rs17156442 | 11:61614023 | t/c | 0.0489 | 0.2323 | 0.2413 | 3.36E-01 | 53776 |
|  | rs6936315 | 6:11035972 | c/t | 0.8198 | 0.0853 | 0.1307 | 5.14E-01 | 63364 |
|  | rs2727266 | 11:61704334 | a/g | 0.9177 | 0.4066 | 0.1904 | 3.27E-02 | 62063 |
|  | rs6928281 | 6:10908917 | g/t | 0.6759 | 0.1523 | 0.1073 | 1.56E-01 | 65775 |
|  | rs7942717 | 11:61647288 | a/g | 0.9134 | 0.1775 | 0.2459 | 4.70E-01 | 44317 |
|  | rs12199131 | 6:10932569 | a/g | 0.2793 | 0.0279 | 0.1118 | 8.03E-01 | 65911 |
|  | **rs174472** | 11:61671956 | / | / | / | / | / | / |
|  | rs7394871 | 11:61652514 | a/c | 0.0620 | 0.4762 | 0.2696 | 7.73E-02 | 42422 |
|  | rs498793 | 11:61624705 | c/t | 0.4060 | 0.0331 | 0.1239 | 7.89E-01 | 41219 |
|  | rs1109748 | 11:61722645 | a/c | 0.0730 | 0.0712 | 0.2230 | 7.50E-01 | 57982 |
|  | rs780094 | 2:27741237 | c/t | 0.4161 | 0.0993 | 0.0984 | 3.13E-01 | 69740 |
|  | rs3734398 | 6:10982973 | c/t | 0.5656 | 0.1252 | 0.0979 | 2.01E-01 | 69076 |
| EPA | rs412334 | 11:61560261 | t/c | 0.1511 | 0.1597 | 0.1729 | 3.56E-01 | 40785 |
|  | rs7394871 | 11:61652514 | a/c | 0.0620 | 0.4762 | 0.2696 | 7.73E-02 | 42422 |
|  | rs498793 | 11:61624705 | c/t | 0.4060 | 0.0331 | 0.1239 | 7.89E-01 | 41219 |
|  | rs1109748 | 11:61722645 | a/c | 0.0730 | 0.0712 | 0.2230 | 7.50E-01 | 57982 |
|  | rs1145652 | 5:164764087 | a/g | 0.8724 | 0.2386 | 0.1470 | 1.04E-01 | 60710 |
|  | rs174538 | 11:61560081 | a/g | 0.2910 | 0.0899 | 0.1051 | 3.92E-01 | 68545 |
|  | rs3798713 | 6:11008622 | c/g | 0.4363 | 0.1282 | 0.0975 | 1.89E-01 | 69617 |

*Regression coefficient associated with one copy of the effect allele.

**Table S14.** Association of n-3 PUFA instrumental SNPs with DBP

| **n-3 PUFA** | **Marker Name** | **Chr. Position** | **EA/NEA** | **EAF** | **Effect*** | **SE** | ***P* value** | **N** |
| --- | --- | --- | --- | --- | --- | --- | --- | --- |
| ALA | rs198464 | 11:61521621 | a/g | 0.4968 | 0.0290 | 0.0616 | 6.38E-01 | 68389 |
|  | rs740006 | 11:61557868 | t/c | 0.8826 | 0.0285 | 0.1399 | 8.39E-01 | 33237 |
|  | rs17762402 | 11:61553201 | g/a | 0.0571 | 0.3187 | 0.1818 | 7.96E-02 | 27188 |
|  | rs412334 | 11:61560261 | t/c | 0.1511 | 0.1606 | 0.1085 | 1.39E-01 | 40698 |
|  | rs174547 | 11:61570783 | c/t | 0.6762 | 0.1739 | 0.0647 | 7.14E-03 | 69789 |
| DHA | rs2236212 | 6:10995015 | c/g | 0.4298 | 0.0835 | 0.0619 | 1.78E-01 | 69551 |
| DPA | rs198464 | 11:61521621 | a/g | 0.4968 | 0.0290 | 0.0616 | 6.38E-01 | 68389 |
|  | rs740006 | 11:61557868 | t/c | 0.8826 | 0.0285 | 0.1399 | 8.39E-01 | 33237 |
|  | rs17762402 | 11:61553201 | g/a | 0.0571 | 0.3187 | 0.1818 | 7.96E-02 | 27188 |
|  | rs412334 | 11:61560261 | t/c | 0.1511 | 0.1606 | 0.1085 | 1.39E-01 | 40698 |
|  | rs174547 | 11:61570783 | c/t | 0.6762 | 0.1739 | 0.0647 | 7.14E-03 | 69789 |
|  | rs10792320 | 11:61746291 | a/c | 0.6608 | 0.0135 | 0.0644 | 8.34E-01 | 68179 |
|  | rs17156442 | 11:61614023 | t/c | 0.0489 | 0.0191 | 0.1556 | 9.02E-01 | 53743 |
|  | rs6936315 | 6:11035972 | c/t | 0.8198 | 0.0211 | 0.0829 | 7.99E-01 | 63429 |
|  | rs2727266 | 11:61704334 | a/g | 0.9177 | 0.1068 | 0.1208 | 3.76E-01 | 62072 |
|  | rs6928281 | 6:10908917 | g/t | 0.6759 | 0.0426 | 0.0681 | 5.32E-01 | 65751 |
|  | rs7942717 | 11:61647288 | a/g | 0.9134 | 0.0842 | 0.1592 | 5.97E-01 | 44140 |
|  | rs12199131 | 6:10932569 | a/g | 0.2793 | 0.0437 | 0.0704 | 5.35E-01 | 65904 |
|  | **rs174472** | 11:61671956 | / | / | / | / | / | / |
|  | rs7394871 | 11:61652514 | a/c | 0.0620 | 0.4301 | 0.1722 | 1.25E-02 | 42518 |
|  | rs498793 | 11:61624705 | c/t | 0.4060 | 0.1049 | 0.0792 | 1.85E-01 | 41167 |
|  | rs1109748 | 11:61722645 | a/c | 0.0730 | 0.1090 | 0.1414 | 4.41E-01 | 57897 |
|  | rs780094 | 2:27741237 | c/t | 0.4161 | 0.0378 | 0.0623 | 5.44E-01 | 69741 |
|  | rs3734398 | 6:10982973 | c/t | 0.5656 | 0.0848 | 0.0622 | 1.72E-01 | 69069 |
| EPA | rs412334 | 11:61560261 | t/c | 0.1511 | 0.1606 | 0.1085 | 1.39E-01 | 40698 |
|  | rs7394871 | 11:61652514 | a/c | 0.0620 | 0.4301 | 0.1722 | 1.25E-02 | 42518 |
|  | rs498793 | 11:61624705 | c/t | 0.4060 | 0.1049 | 0.0792 | 1.85E-01 | 41167 |
|  | rs1109748 | 11:61722645 | a/c | 0.0730 | 0.1090 | 0.1414 | 4.41E-01 | 57897 |
|  | rs1145652 | 5:164764087 | a/g | 0.8724 | 0.1330 | 0.0929 | 1.52E-01 | 60697 |
|  | rs174538 | 11:61560081 | a/g | 0.2910 | 0.1824 | 0.0668 | 6.31E-03 | 68524 |
|  | rs3798713 | 6:11008622 | c/g | 0.4363 | 0.0837 | 0.0619 | 1.77E-01 | 69606 |

*Regression coefficient associated with one copy of the effect allele.

**Table S15.** Association of n-3 PUFA instrumental SNPs with WHR

| **n-3 PUFA** | **Marker Name** | **Chr. Position** | **EA/NEA** | **EAF** | **Effect*** | **SE** | ***P* value** | **N** |
| --- | --- | --- | --- | --- | --- | --- | --- | --- |
| ALA | rs198464 | 11:61521621 | a/g | 0.4739 | -0.0016 | 0.0018 | 3.84E-01 | 626283 |
|  | rs740006 | 11:61557868 | t/c | 0.9016 | 0.0042 | 0.0031 | 1.68E-01 | 653322 |
|  | rs17762402 | 11:61553201 | a/g | 0.0740 | 0.0115 | 0.0034 | 8.05E-04 | 658833 |
|  | rs412334 | 11:61560261 | t/c | 0.1514 | 0.0041 | 0.0024 | 9.23E-02 | 666764 |
|  | rs174547 | 11:61570783 | t/c | 0.6556 | 0.0046 | 0.0018 | 9.53E-03 | 694609 |
| DHA | rs2236212 | 6:10995015 | c/g | 0.4233 | 0.0009 | 0.0018 | 6.38E-01 | 627060 |
| DPA | rs198464 | 11:61521621 | a/g | 0.4739 | -0.0016 | 0.0018 | 3.84E-01 | 626283 |
|  | rs740006 | 11:61557868 | t/c | 0.9016 | 0.0042 | 0.0031 | 1.68E-01 | 653322 |
|  | rs17762402 | 11:61553201 | a/g | 0.0740 | 0.0115 | 0.0034 | 8.05E-04 | 658833 |
|  | rs412334 | 11:61560261 | t/c | 0.1514 | 0.0041 | 0.0024 | 9.23E-02 | 666764 |
|  | rs174547 | 11:61570783 | t/c | 0.6556 | 0.0046 | 0.0018 | 9.53E-03 | 694609 |
|  | rs10792320 | 11:61746291 | a/c | 0.6362 | 0.0013 | 0.0018 | 4.72E-01 | 681333 |
|  | rs17156442 | 11:61614023 | t/c | 0.0475 | -0.0062 | 0.0039 | 1.14E-01 | 677140 |
|  | rs6936315 | 6:11035972 | t/c | 0.8082 | 0.0018 | 0.0023 | 4.49E-01 | 618658 |
|  | rs2727266 | 11:61704334 | a/g | 0.9197 | 0.0074 | 0.0034 | 3.08E-02 | 627115 |
|  | rs6928281 | 6:10908917 | t/g | 0.6932 | -0.0010 | 0.0019 | 5.92E-01 | 626024 |
|  | rs7942717 | 11:61647288 | a/g | 0.9276 | 0.0008 | 0.0032 | 8.06E-01 | 679459 |
|  | rs12199131 | 6:10932569 | a/g | 0.2792 | -0.0002 | 0.0020 | 9.20E-01 | 626723 |
|  | rs174472 | 11:61671956 | a/g | 0.5684 | 0.0014 | 0.0018 | 4.36E-01 | 650999 |
|  | rs7394871 | 11:61652514 | a/c | 0.0628 | -0.0042 | 0.0037 | 2.57E-01 | 612758 |
|  | rs498793 | 11:61624705 | t/c | 0.3935 | 0.0001 | 0.0019 | 9.43E-01 | 678637 |
|  | rs1109748 | 11:61722645 | a/c | 0.0788 | -0.0025 | 0.0031 | 4.11E-01 | 688936 |
|  | rs780094 | 2:27741237 | t/c | 0.3796 | 0.0052 | 0.0018 | 2.92E-03 | 694580 |
|  | rs3734398 | 6:10982973 | t/c | 0.5700 | -0.0006 | 0.0018 | 7.35E-01 | 626982 |
| EPA | rs412334 | 11:61560261 | t/c | 0.1514 | 0.0041 | 0.0024 | 9.23E-02 | 666764 |
|  | rs7394871 | 11:61652514 | a/c | 0.0628 | -0.0042 | 0.0037 | 2.57E-01 | 612758 |
|  | rs498793 | 11:61624705 | t/c | 0.3935 | 0.0001 | 0.0019 | 9.43E-01 | 678637 |
|  | rs1109748 | 11:61722645 | a/c | 0.0788 | -0.0025 | 0.0031 | 4.11E-01 | 688936 |
|  | rs1145652 | 5:164764087 | a/g | 0.8571 | 0.0083 | 0.0026 | 1.62E-03 | 626874 |
|  | rs174538 | 11:61560081 | a/g | 0.3169 | -0.0042 | 0.0018 | 2.34E-02 | 694497 |
|  | rs3798713 | 6:11008622 | c/g | 0.4299 | 0.0015 | 0.0018 | 3.95E-01 | 627081 |

*Regression coefficient associated with one copy of the effect allele.

**Table S16.** Association of n-3 PUFA instrumental SNPs with BMI

| **n-3 PUFA** | **Marker Name** | **Chr. Position** | **EA/NEA** | **EAF** | **Effect*** | **SE** | ***P* value** | **N** |
| --- | --- | --- | --- | --- | --- | --- | --- | --- |
| ALA | rs198464 | 11:61521621 | a/g | 0.4740 | 0.0040 | 0.0017 | 1.79E-02 | 718514 |
|  | rs740006 | 11:61557868 | t/c | 0.9013 | 0.0040 | 0.0029 | 1.74E-01 | 763282 |
|  | rs17762402 | 11:61553201 | a/g | 0.0740 | 0.0030 | 0.0033 | 3.60E-01 | 751575 |
|  | rs412334 | 11:61560261 | t/c | 0.1511 | -0.0014 | 0.0023 | 5.32E-01 | 769149 |
|  | rs174547 | 11:61570783 | t/c | 0.6554 | -0.0029 | 0.0017 | 9.15E-02 | 806740 |
| DHA | rs2236212 | 6:10995015 | c/g | 0.4238 | 0.0001 | 0.0017 | 9.66E-01 | 718500 |
| DPA | rs198464 | 11:61521621 | a/g | 0.4740 | 0.0040 | 0.0017 | 1.79E-02 | 718514 |
|  | rs740006 | 11:61557868 | t/c | 0.9013 | 0.0040 | 0.0029 | 1.74E-01 | 763282 |
|  | rs17762402 | 11:61553201 | a/g | 0.0740 | 0.0030 | 0.0033 | 3.60E-01 | 751575 |
|  | rs412334 | 11:61560261 | t/c | 0.1511 | -0.0014 | 0.0023 | 5.32E-01 | 769149 |
|  | rs174547 | 11:61570783 | t/c | 0.6554 | -0.0029 | 0.0017 | 9.15E-02 | 806740 |
|  | rs10792320 | 11:61746291 | a/c | 0.6357 | -0.0045 | 0.0017 | 7.47E-03 | 792640 |
|  | rs17156442 | 11:61614023 | t/c | 0.0472 | 0.0070 | 0.0037 | 6.03E-02 | 781955 |
|  | rs6936315 | 6:11035972 | t/c | 0.8083 | 0.0037 | 0.0022 | 9.28E-02 | 709160 |
|  | rs2727266 | 11:61704334 | a/g | 0.9185 | -0.0054 | 0.0033 | 9.57E-02 | 718043 |
|  | rs6928281 | 6:10908917 | t/g | 0.6934 | 0.0030 | 0.0019 | 1.02E-01 | 718548 |
|  | rs7942717 | 11:61647288 | a/g | 0.9285 | -0.0042 | 0.0031 | 1.73E-01 | 789751 |
|  | rs12199131 | 6:10932569 | a/g | 0.2794 | 0.0021 | 0.0019 | 2.81E-01 | 718077 |
|  | rs174472 | 11:61671956 | a/g | 0.5680 | -0.0013 | 0.0017 | 4.58E-01 | 743176 |
|  | rs7394871 | 11:61652514 | a/c | 0.0621 | 0.0054 | 0.0035 | 1.23E-01 | 703035 |
|  | rs498793 | 11:61624705 | t/c | 0.3939 | 0.0015 | 0.0018 | 4.09E-01 | 791648 |
|  | rs1109748 | 11:61722645 | a/c | 0.0780 | 0.0007 | 0.0029 | 8.17E-01 | 801236 |
|  | rs780094 | 2:27741237 | t/c | 0.3798 | -0.0096 | 0.0016 | **5.85E-09** | 806724 |
|  | rs3734398 | 6:10982973 | t/c | 0.5699 | 0.0003 | 0.0017 | 8.82E-01 | 718415 |
| EPA | rs412334 | 11:61560261 | t/c | 0.1511 | -0.0014 | 0.0023 | 5.32E-01 | 769149 |
|  | rs7394871 | 11:61652514 | a/c | 0.0621 | 0.0054 | 0.0035 | 1.23E-01 | 703035 |
|  | rs498793 | 11:61624705 | t/c | 0.3939 | 0.0015 | 0.0018 | 4.09E-01 | 791648 |
|  | rs1109748 | 11:61722645 | a/c | 0.0780 | 0.0007 | 0.0029 | 8.17E-01 | 801236 |
|  | rs1145652 | 5:164764087 | a/g | 0.8561 | 0.0021 | 0.0025 | 3.93E-01 | 718423 |
|  | rs174538 | 11:61560081 | a/g | 0.3179 | 0.0024 | 0.0017 | 1.71E-01 | 806700 |
|  | rs3798713 | 6:11008622 | c/g | 0.4301 | -0.0001 | 0.0017 | 9.58E-01 | 718530 |

*Regression coefficient associated with one copy of the effect allele.

**Table S17.** Results of MR analyses testing causal effect of n-3 PUFA on IHD and cardiometabolic risk factors

| **n-3 PUFA** | **Outcome** | **No of IVs** | **IVW** | | **WM** | | **MR-Egger** | | | | **MR-PRESSO** | | |
| --- | --- | --- | --- | --- | --- | --- | --- | --- | --- | --- | --- | --- | --- |
|  |  |  | **CE (SE)** | ***p*** | **CE (SE)** | ***p*** | **CE (SE)** | ***p*** | **CE int (SE int)** | ***p*_int_** | **Nooutl** | **CE (SD)** | ***p*** |
| ALA | IHD | 4 | -1.173(0.531) | **0.027** | -1.890(0.691) | **0.006** | -0.246(1.800) | 0.891 | -0.012(0.020) | 0.565 | 0 | -1.173(0.708) | 0.196 |
|  | MI | 4 | -0.841(0.705) | 0.233 | -1.045(0.810) | 0.197 | -0.807(1.553) | 0.604 | -0.001(0.017) | 0.980 | 0 | -0.841(0.400) | 0.126 |
|  | T2D | 4 | -2.059(0.762) | **0.007** | -1.758(0.917) | 0.055 | 0.085(1.779) | 0.962 | -0.028(0.021) | 0.182 | 0 | -2.059(0.589) | **0.040** |
|  | HDL | 4 | -0.915(0.235) | **< 0.001** | -1.521(0.345) | **< 0.001** | -0.410(1.695) | 0.809 | -0.007(0.021) | 0.746 | 1 | -1.573(0.057) | **0.001** |
|  | LDL | 4 | -0.937(0.250) | **< 0.001** | -1.276(0.411) | **0.002** | 0.473(2.027) | 0.816 | -0.019(0.025) | 0.450 | 2 | -1.356(0.375) | 0.172 |
|  | TC | 4 | -0.894(0.240) | **< 0.001** | -1.144(0.417) | **0.006** | 0.607(1.775) | 0.732 | -0.020(0.022) | 0.359 | 2 | -1.322(0.497) | 0.229 |
|  | TG | 4 | -0.715(0.225) | **0.002** | -1.521(0.346) | **< 0.001** | 0.747(2.446) | 0.760 | -0.020(0.030) | 0.517 | 0 | -0.715(0.852) | 0.463 |
|  | SBP | 4 | -7.296(7.387) | 0.323 | -9.072(9.124) | 0.320 | -8.264(9.911) | 0.678 | 0.011(0.204) | 0.957 | 0 | -7.296(7.364) | 0.395 |
|  | DBP | 4 | -8.106(4.744) | 0.088 | -8.733(5.618) | 0.120 | -10.254(11.885) | 0.388 | 0.024(0.121) | 0.839 | 0 | -8.106(4.423) | 0.164 |
|  | BMI | 4 | -0.021(0.099) | 0.831 | 0.064(0.130) | 0.625 | 0.412(0.347) | 0.235 | -0.006(0.004) | 0.168 | 0 | -0.021(0.168) | 0.908 |
|  | WHR | 4 | -0.185(0.104) | 0.077 | -0.257(0.149) | 0.085 | -0.529(0.588) | 0.369 | 0.004(0.007) | 0.518 | 0 | -0.185(0.224) | 0.470 |
| DPA | IHD | 15 | 0.147(0.077) | 0.056 | 0.067(0.113) | 0.553 | -0.134(0.188) | 0.476 | 0.011(0.007) | 0.095 | 0 | 0.147(0.087) | 0.114 |
|  | MI | 15 | -0.021(0.099) | 0.837 | -0.017(0.136) | 0.904 | 0.193(0.230) | 0.402 | -0.008(0.008) | 0.303 | 0 | -0.021(0.080) | 0.800 |
|  | T2D | 15 | 0.423(0.115) | **< 0.001** | 0.372(0.155) | **0.016** | 0.117(0.258) | 0.649 | 0.013(0.009) | 0.184 | 0 | 0.423(0.104) | **0.001** |
|  | HDL | 15 | 0.079(0.037) | **0.032** | 0.133(0.070) | 0.060 | -0.097(0.215) | 0.652 | 0.008(0.008) | 0.354 | 5 | 0.112(0.076) | 0.174 |
|  | LDL | 15 | 0.135(0.039) | **0.001** | 0.245(0.085) | **0.004** | -0.141(0.215) | 0.512 | 0.012(0.008) | 0.148 | 6 | 0.232(0.086) | **0.028** |
|  | TC | 15 | 0.109(0.038) | **0.004** | 0.222(0.080) | **0.006** | -0.106(0.217) | 0.625 | 0.009(0.008) | 0.263 | 6 | 0.209(0.071) | **0.018** |
|  | TG | 15 | 0.051(0.036) | 0.154 | 0.062(0.085) | 0.468 | -0.277(0.242) | 0.251 | 0.014(0.009) | 0.127 | 8 | 0.128(0.093) | 0.218 |
|  | SBP | 14 | 0.413(1.052) | 0.695 | 0.593(1.555) | 0.703 | -2.837(2.672) | 0.288 | 0.126(0.093) | 0.175 | 0 | 0.413(1.216) | 0.739 |
|  | DBP | 14 | 0.364(0.672) | 0.587 | 0.853(1.018) | 0.402 | -0.674(1.811) | 0.710 | 0.040(0.063) | 0.523 | 0 | 0.364(0.776) | 0.647 |
|  | BMI | 15 | -0.011(0.015) | 0.458 | -0.019(0.021) | 0.350 | -0.053(0.051) | 0.299 | 0.002(0.002) | 0.360 | 0 | -0.011(0.023) | 0.629 |
|  | WHR | 15 | 0.043(0.016) | **0.006** | 0.057(0.024) | **0.019** | 0.074(0.044) | 0.088 | -0.001(0.002) | 0.426 | 0 | 0.043(0.019) | **0.041** |
| EPA | IHD | 6 | 0.039(0.104) | 0.707 | -0.017(0.145) | 0.908 | -0.491(0.383) | 0.200 | 0.025(0.017) | 0.145 | 0 | 0.039(0.133) | 0.780 |
|  | MI | 6 | 0.086(0.134) | 0.521 | 0.192(0.179) | 0.284 | -0.308(0.437) | 0.481 | 0.018(0.019) | 0.344 | 0 | 0.086(0.126) | 0.526 |
|  | T2D | 6 | 0.077(0.160) | 0.631 | 0.076(0.212) | 0.719 | 0.202(0.665) | 0.762 | -0.006(0.029) | 0.844 | 0 | 0.077(0.182) | 0.690 |
|  | HDL | 6 | 0.026(0.055) | 0.640 | 0.055(0.099) | 0.578 | -0.882(0.535) | 0.099 | 0.039(0.022) | 0.079 | 4 | 0.023(0.013) | 0.325 |
|  | LDL | 6 | 0.175(0.059) | **0.003** | 0.267(0.113) | **0.018** | -1.126(0.584) | 0.054 | 0.056(0.024) | **0.021** | 4 | 0.180(0.041) | 0.142 |
|  | TC | 6 | 0.120(0.057) | **0.034** | 0.298(0.111) | **0.007** | -1.139(0.645) | 0.077 | 0.054(0.027) | **0.044** | 4 | 0.105(0.114) | 0.526 |
|  | TG | 6 | 0.079(0.053) | 0.130 | 0.250(0.083) | **0.003** | -1.090(0.408) | **0.008** | 0.050(0.017) | **0.003** | 4 | 0.194(0.099) | 0.300 |
|  | SBP | 6 | 0.994(1.408) | 0.480 | 2.430(1.920) | 0.206 | -10.258(4.696) | **0.029** | 0.497(0.198) | **0.012** | 0 | 0.994(1.782) | 0.601 |
|  | DBP | 6 | 0.708(0.894) | 0.428 | 2.489(1.255) | 0.047 | -9.247(2.996) | **0.002** | 0.439(0.126) | **< 0.001** | 1 | 2.276(0.935) | 0.072 |
|  | BMI | 6 | -0.012(0.020) | 0.549 | -0.014(0.026) | 0.590 | -0.112(0.063) | 0.075 | 0.005(0.003) | 0.094 | 0 | -0.012(0.018) | 0.525 |
|  | WHR | 6 | 0.062(0.022) | **0.004** | 0.046(0.027) | 0.089 | 0. 035(0.089) | 0.695 | 0.001(0.004) | 0.745 | 0 | 0.062(0.026) | 0.063 |
| DHA | IHD | 1 | -0.011(0.072) | 0.884 | / | / | / | / | / | / | / | / | / |
|  | MI | 1 | -0.055(0.091) | 0.547 | / | / | / | / | / | / | / | / | / |
|  | T2D | 1 | 0.067(0.115) | 0.559 | / | / | / | / | / | / | / | / | / |
|  | HDL | 1 | -0.004(0.042) | 0.934 | / | / | / | / | / | / | / | / | / |
|  | LDL | 1 | -0.049(0.046) | 0.290 | / | / | / | / | / | / | / | / | / |
|  | TC | 1 | -0.011(0.045) | 0.814 | / | / | / | / | / | / | / | / | / |
|  | TG | 1 | -0.035(0.042) | 0.395 | / | / | / | / | / | / | / | / | / |
|  | SBP | 1 | -1.087(0.862) | 0.208 | / | / | / | / | / | / | / | / | / |
|  | DBP | 1 | -0.738(0.547) | 0.177 | / | / | / | / | / | / | / | / | / |
|  | BMI | 1 | -0.001(0.015) | 0.953 | / | / | / | / | / | / | / | / | / |
|  | WHR | 1 | -0.008(0.016) | 0.617 | / | / | / | / | / | / | / | / | / |
